# Human Brain Microvascular Endothelial Cells Exposure to SARS-CoV-2 Leads to Inflammatory Activation through NF-κB Non-Canonical Pathway and Mitochondrial Remodeling

**DOI:** 10.3390/v15030745

**Published:** 2023-03-14

**Authors:** Carolline Soares Motta, Silvia Torices, Barbara Gomes da Rosa, Anne Caroline Marcos, Liandra Alvarez-Rosa, Michele Siqueira, Thaidy Moreno-Rodriguez, Aline da Rocha Matos, Braulia Costa Caetano, Jessica Santa Cruz de Carvalho Martins, Luis Gladulich, Erick Loiola, Olivia R. M. Bagshaw, Jeffrey A. Stuart, Marilda M. Siqueira, Joice Stipursky, Michal Toborek, Daniel Adesse

**Affiliations:** 1 Laboratório de Biologia Estrutural, Instituto Oswaldo Cruz, Fiocruz, Rio de Janeiro 21040-360, Brazil; 2 Department of Biochemistry and Molecular Biology, University of Miami Miller School of Medicine, Miami, FL 33136, USA; 3 Laboratório Compartilhado, Instituto de Ciências Biomédicas, UFRJ, Rio de Janeiro 05508-000, Brazil; 4 Urology Department, University of California San Francisco, San Francisco, CA 94143, USA; 5 Helen Diller Family Comprehensive Cancer Center, University of California San Francisco, San Francisco, CA 94143, USA; 6 Laboratório de Vírus Respiratórios, Exantemáticos, Enterovírus e Emergências Virais (LVRE), Instituto Oswaldo Cruz, Fiocruz, Rio de Janeiro 21040-360, Brazil; 7 Faculty of Mathematics & Science, Brock University, St. Catharines, ON L2S 3A1, Canada

**Keywords:** COVID-19, blood–brain barrier, mitochondrial dynamics, NF-κB signaling pathway, endothelial activation

## Abstract

Neurological effects of COVID-19 and long-COVID-19, as well as neuroinvasion by SARS-CoV-2, still pose several questions and are of both clinical and scientific relevance. We described the cellular and molecular effects of the human brain microvascular endothelial cells (HBMECs*)* in vitro exposure by SARS-CoV-2 to understand the underlying mechanisms of viral transmigration through the blood–brain barrier. Despite the low to non-productive viral replication, SARS-CoV-2-exposed cultures displayed increased immunoreactivity for cleaved caspase-3, an indicator of apoptotic cell death, tight junction protein expression, and immunolocalization. Transcriptomic profiling of SARS-CoV-2-challenged cultures revealed endothelial activation via NF-κB non-canonical pathway, including RELB overexpression and mitochondrial dysfunction. Additionally, SARS-CoV-2 led to altered secretion of key angiogenic factors and to significant changes in mitochondrial dynamics, with increased mitofusin-2 expression and increased mitochondrial networks. Endothelial activation and remodeling can further contribute to neuroinflammatory processes and lead to further BBB permeability in COVID-19.

## 1. Introduction

Coronavirus disease 2019 (COVID-19), caused by infection with severe acute respiratory syndrome-related coronavirus 2 (SARS-CoV-2), remains a major health threat globally. The USA continues to lead the world with a total of 102 million COVID-19 cases and 1.1 million deaths by the end of January 2023 [1]. Even though vaccines, which mostly prevent serious illness and death, have been widely available in the U.S. and many countries, only 59% of the overall population is fully vaccinated. This is below the estimated 85–90% threshold assumed to be needed to stop the spread of SARS-CoV-2 and make the virus endemic. Globally, COVID-19 cases continue rising, and new variants and subvariants (e.g., omicron BA.1, BA.2 [BA.2.12, BA.2.12.1]) were recently identified in different countries, spreading globally [2].

SARS-CoV-2 is a member of the family of β-coronaviruses, similar to two other highly pathogenic coronaviruses, severe acute respiratory syndrome coronavirus (SARS-CoV) and Middle East respiratory syndrome coronavirus (MERS-CoV). Initial SARS-CoV-2 infection investigated cases led to the isolation of the virus in human respiratory epithelial cells [3,4] and its genome sequencing deposited (GISAID accession IDs: EPI_ISL_402119, 402120 and 402121). SARS-CoV-2 is an enveloped, positive-sense, and single-stranded RNA virus. Its genome encodes non-structural proteins (such as 3-chymotrypsin-like protease, papain-like protease, helicase, and RNA-dependent RNA polymerase; all key enzymes in the viral life cycle), structural proteins (spike [S] protein, membrane [M] protein, envelope [E] protein, and nucleocapsid [N] protein), and accessory proteins. 

It is now known that SARS-CoV-2 interacts with and infects human cells through the ligation of the S1 subunit of the S protein with host cell receptors, especially the angiotensin-2 converting enzyme (ACE2) that serves as an entry receptor to the virus, representing its main route of entry into the host cell [5]. Pulmonary, cardiac, and intestinal epithelia and endothelial cells express high levels of ACE-2 [3]. Upon S1-ACE2 interaction, a transmembrane serine protease 2 (TMPRSS2) is required for priming the S protein and viral entry into the cell [4,6,7]. Along with ACE2 and TMPRSS2, several other proteins have been suggested to participate in SARS-CoV-2 entry into human cells, such as ADAM metallopeptidase domain 17 (ADAM17) [8,9], dipeptidyl peptidase 4 (DPP4) [10,11], angiotensin II receptor type 2 (AGTR2) [12,13], basigin (BSG, also called extracellular matrix metalloproteinase inducer [EMMPRIN] or cluster of differentiation 147 [CD147]) [14,15], aminopeptidase N (ANPEP) [16], and cathepsin B/L [5,17].

Among the most commonly observed symptoms in COVID-19 patients, alterations of neural functions are frequently detected, from mild cases with loss of taste and smell, dizziness, and headaches, to more extreme cases with the occurrence of acute cerebrovascular disease, including episodes of vascular encephalic accidents, loss of consciousness, ataxia, and epilepsy [18]. The Central Nervous System (CNS)-related symptoms are also prominent for so-called chronic or long COVID. The CNS is a well-documented target of β-coronavirus infections, such as SARS-CoV-2, and to date, several studies detected SARS-CoV-2 in the brain and the cerebrospinal fluid of COVID-19 patients [19,20,21,22]. Distinct routes of SARS-CoV-2 entry into the brain have been proposed, such as the olfactory nerve [23,24,25], the choroid plexus, and the blood–brain barrier (BBB) [26]. The BBB represents a physiological interphase between systemic blood circulation and the brain parenchyma. The BBB is primarily formed by endothelial cells that are surrounded by astrocytes, neurons, pericytes, and microglia cells that, by coordinating functions with endothelial cells, form the structural elements of the BBB known as the neuro-vascular unit [27]. However, the contribution of the BBB may be particularly important due to the presence of the virus in the bloodstream allowing the passage of viral particles through the wall of brain capillaries to brain parenchyma. While the mechanisms of SARS-CoV-2 neuroinvasion are not fully understood, it has been suggested that infection of BBB capillary-composing cells could be critical to triggering CNS impairment [28,29]. Among the NVU-forming cells, endothelial cells are especially important for ensuring BBB function, and several studies have recently described EC as key players in SARS-CoV-2-induced pathogenesis [30,31,32].

Several reports have correlated the infection outcome with vascular dysfunction, establishing vascular inflammation and cytokine storms promoted by immune responses as critical factors contributing to the worsening of the clinical condition and even death. Endothelial dysfunction may have important consequences, which include ischemia, altered angiogenesis and coagulation, inflammation, and tissue edema. Therefore, COVID-19-related endothelitis could explain the systemic microcirculatory dysfunction observed in patients, including a chronic form of this disease. It was previously demonstrated that the treatment of human brain microvascular endothelial cells with recombinant S1 protein resulted in the endothelial permeability and altered the levels of pro-inflammatory cytokines [33]. However, little is known about the involvement of brain microvasculature in brain infection by SARS-CoV-2, which may result in endothelial activation and hyper-inflammatory responses. Even less is known if damages to the BBB could be propagated to neural tissue and, therefore, be the triggering mechanism of neural abnormalities that promote neurological symptoms observed in COVID-19 patients.

In the present work, we describe the cellular and molecular effects of HBMEC exposed to SARS-CoV-2 in order to gain insight into possible routes by which the virus affects the BBB and invades the brain parenchyma. HBMECs susceptibility to infection was compared to that of gold-standard African monkey kidney epithelial Vero cells, including viral production and activation of caspase-3. Further characterization of SARS-CoV-2 effects on HBMECs was performed by two unbiased analyses of gene expression and angiogenic factor secretion. Transcriptomic analyses revealed activation of noncanonical NF-κB signaling pathway and changes in mitochondrial quality control, with increased mitochondrial networks and mitofusin-2 expression in SARS-CoV-2-challenged cultures. Our data demonstrate that exposure to SARS-CoV-2 leads to brain endothelium activation, thus contributing to promoting increased neuroinflammation in Neuro-COVID-19. 

## 2. Methods

### 2.1. Cell Culture

Human brain endothelial cells (HBMECs) were a gift from Prof. Dennis Grab (Department of Pathology, Johns Hopkins School of Medicine). Cells were immortalized using an SV40-LT plasmid [34] and were maintained in 199 medium with 10% fetal bovine serum (FBS) and 1% antibiotics (penicillin/streptomycin, ThermoFisher, Carlsbad, CA, USA) up to passage 38. Vero E6 cells (African green monkey kidney epithelial cells) were used as the gold standard for viral isolation and propagation and were used in a few experiments as a positive control for efficient SARS-CoV-2 infection. Vero E6 cells culture medium consisted of Dulbecco’s Modified Eagle Medium (DMEM, ThermoFisher, Carlsbad, CA, USA) formulated with D-glucose (4.5 g/L, Sigma-Aldrich, St. Louis, MO, USA), L-Glutamine (3.9 mM, Sigma-Aldrich, St. Louis, MO, USA) supplemented with 100× penicillin-streptomycin solution (to final 100 U/mL and 100 μg/mL, respectively, ThermoFisher, Carlsbad, CA, USA), and inactivated FBS (USDA-qualified region FBS) at 10%. Both cell and viral cultures were incubated at 37°C and 5% CO_2_ atmosphere.

### 2.2. SARS-CoV-2 Isolate

All the procedures associated with the viral isolation and further infection assays were performed in a biosafety level-3 laboratory in accordance with the WHO guidelines [35]. The SARS-CoV-2 isolate used in this study was previously obtained from a nasopharyngeal swab sample collected from a COVID-19 patient diagnosed at Fiocruz COVID-19 regional reference center for WHO, in March 2020, in Brazil, as part of the Brazilian Ministry of Health surveillance system. The clinical sample was recovered from a patient that developed a mild disease and fully recovered. Viral isolation was performed in Vero E6 cells, as previously described [36]. In addition, the isolate was characterized by transmission electron microscopy [37]. The viral titer of the isolate was increased by an additional passage in Vero E6 cells to obtain a working stock. The 50% Tissue Culture Infectious Dose (TCID_50_) titer of the viral working stock was determined by limiting dilution and infection of Vero E6 cells. Genetic characterization of the isolate was performed by whole-genome sequencing, and its genome is available in the Global initiative on sharing all influenza data (GISAID) under the accession numbers EPI_ISL ID 427294 (https://www.epicov.org/ accessed on 10 August 2020), confirming its classification as the original Wuhan strain (Pango lineage B.1.1.33). All procedures involving patient samples were approved by the Committee of Ethics in Human Research of the Oswaldo Cruz Institute (registration number CAAE 68118417.6.0000.5248).

### 2.3. SARS-CoV-2 Challenge

Cells were previously cultured to obtain confluent monolayers for the moment of infection. After that, cells were washed once with PBS and further incubated with SARS-CoV-2 inoculums diluted in non-supplemented DMEM or medium 199, corresponding to indicated multiplicities of infection (MOI) for one hour. After that, inoculums were removed from cells and replaced by their appropriate supplemented medium with N-tosyl-L-phenylalanine chloromethyl ketone (TPCK)-treated trypsin (Sigma-Aldrich, St. Louis, MO, USA) at 1 µg/mL.

### 2.4. Viral Quantification

We evaluated the SARS-CoV-2 replication of infected cell cultures over time by measuring the number of viral RNA copies in their culture media over time. Viral RNA was extracted from 140 μL of cell-free culture media via QIAamp Viral RNA mini kit (Qiagen, Hilden, Germany) according to the manufacturer’s instructions. Reverse transcription and SARS-CoV-2 gene amplification were performed in one-step reactions with a quantitative real-time PCR kit developed by Biomanguinhos Institute (Fiocruz, Rio de Janeiro, Brazil) in an ABI 7500 thermocycler (Applied Biosystems, Carlsbad, CA, USA). As a quantification standard, we used a SARS-CoV-2 plasmid control containing the reference sequence of the viral envelope (E) gene with a known number of copies (IDT, Newark, NJ, USA). Therefore, a concentration curve was prepared by performing serial dilutions of the plasmid.

### 2.5. RNA Libraries and Sequencing (RNA-Seq)

For RNA-Seq analysis, three independent replicates were prepared for each treatment group, Mock, MOI 0.01-, and MOI 0.1-exposed HBMEC cultures, after 6 and 24 h. Total RNA was isolated via the miRNeasy micro kit (Qiagen, Hilden, Germany) according to the manufacturer’s instructions. The RNA was quantified by O.D. measurement before being assessed for quality by chip-based capillary electrophoresis using Agilent 2100 Bioanalyzer RNA 6000 Pico assays (Agilent Technologies, St Clara, CA, USA; Part # 5067-1513).

Libraries were prepared from 150 nanograms (ng) of DNA-free total RNA using the Universal Plus mRNA-Seq Library Prep Kit (NuGEN Technologies, Inc., San Francisco, CA, USA; Part # 0508-96). The quality and size distribution of the amplified libraries was determined by chip-based capillary electrophoresis on Agilent 2100 Bioanalyzer High Sensitivity DNA assays (Agilent Technologies; Part # 5067-4626). Libraries were quantified using the Takara Library Quantification Kit (Shiga, Japan; Part # 638324). The libraries were pooled at equimolar concentrations and diluted prior to loading onto a P3 flow cell (Illumina, San Diego, CA, USA; Part # 20027800) with the P3 300 Cycle reagent kit (Illumina, San Diego, CA; Part # 20038732) on the NextSeq2000 instrument (Illumina, San Diego, CA, USA).

### 2.6. RNA-Seq Data Analysis

Reads: R1 and R2 were trimmed 12 nucleotides (nt) to remove low-quality sequences. Bases with a quality score of less than Q20 were trimmed off the right end of each R1 and R2. Illumina adapter sequences were trimmed from the 3′-end of both R1 and R2 reads. Read pairs, in which the mate in the pair was less than 30 nt after trimming, were discarded. These quality-filtered reads were then used for alignment.

Sequence alignment was performed using HISAT2 [38] version 2.0.5 with the following settings:

hisat2 --end-to-end -N 1 -L 20 -i S,1,0.5 -D 25 -R 5 --pen-noncansplice 12 --mp 6,3 --sp 3,0 --time --reorder --known-splicesite-infile [SPLICESITES] --novel-splicesite-outfile splicesites.novel.txt --novel-splicesite-infile splicesites.novel.txt -q –x [hsa38 HISAT2 INDEX] -1 [FASTQ1] -2 [FASTQ2] -S [SAMOUT]. The read summarization program featureCounts [39] version 1.5.1 was used for exon- and gene-level counting. An Ensembl human version 83 GTF file (downloaded from Ensembl Biomart on 22 January 2016) was used for the determination of exon boundaries and the exon–gene relationship during counting. The summarization level used for exon and gene counting was the feature and the meta-feature, respectively. The feature-Counts is available in the Subread package at http://subread.sourceforge.net, accessed on 20 November 2021 [39].

To determine differential gene expression and due to the low coefficient of biological variation, paired comparisons were performed between the untreated control (UC) and MOIs 0.01- and 0.1-treated HBMEC cells at the 6 and 24 h timepoints, using an additive linear model with the untreated group as the blocking factor. Differential gene expression analysis was performed using the EdgeR R package [40]). 

The top differentially expressed genes have consistent UC vs. MOI 0.1 changes for the three replicates at 5% FDR, and an absolute log2 fold change of 0.6 was considered a cut-off to generate the DEG list. Computed z-scores of significant genes are represented in the heatmap. Heatmap was plotted using the ComplexHeatmap R package [41].

### 2.7. Downstream RNA-Seq Analysis

We used a list of genes differentially expressed between MOI 0.1 SARS-CoV-2-exposed and untreated HBMEC cells. The pathway enrichment and interaction networks analysis were performed using clusterProfiler and gprofiler2 R packages [42,43]. Overlapping gene sets from reactome pathway terms were visualized as a chord plot using the GOplot R [44].

### 2.8. RT-qPCR

Cells were grown in 60 mm^2^ dishes, and total RNA was extracted with Trizol reagent (ThermoFisher, Carlsbad, CA, USA), according to the manufacturer’s instructions. One microgram of total RNA was reversely transcribed into cDNA via the SuperScript III system (ThermoFisher, Carlsbad, CA, USA), and 0.5 µL of cDNA was used per RT-qPCR reaction with Power SYBR Green (ThermoFisher, Carlsbad, CA, USA) master mix. Reactions were read in 7500 StepOne Plus from the Oswaldo Cruz Institute. Primer sequences for Drp1, Fis1, ZO-1, claudin-5, HIF-1α, Mfn2, MFF, and TOMM20 are provided in Table S1. For the E gene and Spike1 RT-qPCR, we used the protocols described in [45] and [46], respectively. For the remaining genes, 100 ng of total RNA was used for Taqman reactions using primer probes from ThermoFisher (Carlsbad, CA, USA): Hs00242739_m1 (LTB); Hs00174128_m1 (TNF); Hs00232399_m1 (RELB); Hs00357891_s1 (JUNB); Hs00759776_s1 (FOSL1); Hs00765730_m1 (NFKB1); Hs00174103_m1 (CXCL8); Hs00601975_m1 (CXCL2); Hs00236937_m1 (CXCL1); Hs00173615_m1 (PTX3); Hs00174961_m1 (EDN1); Hs00299953_m1 (SERPINE2); and Hs01028889_g1 (NFKB2). GAPDH (Hs02786624_g1) was used for sample normalization. Gene expression variations were assessed by the 2^ΔΔCt^ method, with Ct as the cycle number at the threshold. Desired PCR result specificity was determined based on melting curve evaluation.

### 2.9. Western Blotting

HBMECs were cultivated in 60 mm^2^ dishes and, at desired times, were washed with PBS and lysed in the presence of 1× Laemmli Buffer (0.0625M Tris, 0.07M SDS, 10% glycerol, 5% β-mercaptoethanol, and bromophenol blue). Protein concentration was measured with BCA Protein Assay Kit according to the manufacturer’s instructions (Thermo Fisher Scientific, Carlsbad, CA, USA). Then, 30 µg of protein were loaded onto 4–20% gradient acrylamide gels (Bio-Rad Laboratories, Hercules, CA, USA). Membranes were blocked with bovine serum album (BSA) 5% in TBS-0.05% Tween20 and incubated overnight at 4 °C with the primary antibodies at 1:1000 dilution in TBST (Table S2). The next day, blots were washed with TBS-0.05% Tween20, incubated for 1 h at room temperature with secondary antibodies (Lincoln, NE, USA), and analyzed using the Licor CLX imaging system and the Image Studio 4.0 software (LI-COR, NE, USA).

### 2.10. Immunofluorescence

Cells were grown on 13-mm round glass coverslips and fixed at desired times with 4% paraformaldehyde in PBS for 10 min at 20 °C, permeabilized with 0.5% Triton x-100 (Sigma–Aldrich, St. Louis, MO, USA), blocked with 3% bovine serum albumin (BSA, Sigma-Aldrich, St. Louis, MO, USA), and incubated overnight with primary antibodies at 4 °C. Cells were washed with PBS and incubated with fluorescently labeled secondary antibodies for 1 h at 37 °C. For nuclear visualization, cells were incubated with DAPI (4′,6-diamidino-2-phenylindole) and mounted in a solution of glycerol and DABCO (1,4-diazabicyclo [2.2.2]octane, Sigma-Aldrich, St. Louis, MO, USA) in PBS. The list of primary and secondary antibodies used in this study is detailed in Table S2.

### 2.11. Quantitative Analysis of Mitochondrial Network Morphology

Mitochondrial network morphology was analyzed using the Mitochondrial Network Analysis Tool (MiNA) for the Fiji distribution of ImageJ [47]. Images were cropped into individual cells. To enhance contrast and sharped mitochondrial images, several pre-processing tools were applied to each image prior to MiNA analysis. First, an unsharp mask (sigma = 3) was used to sharpen images by subtracting a blurred version of the image (i.e., unsharp mask) from the image. The unsharp mask is created by Gaussian blurring the original image and multiplying the blurred image by the mask weight (0.8). Second, a median filter (radius = 1) was applied to each image. The median filter functions by replacing each pixel with the neighborhood median, where the neighborhood size is determined by the radius. Following pre-processing, images underwent thresholding using the Otsu thresholding to produce a binary image [48]. The *mitochondrial footprint* is calculated as the total number of mitochondria-signal positive pixels from the binarized image. A morphological skeleton is then produced from the binarized image using the Skeletonize 2D/3D plug-in [49,50]. This method employs iterative thinning to create a skeleton of mitochondrial structures, one pixel wide. Length measurements of the mitochondrial structures are then measured using the Analyze Skeleton plug-in, resulting in two additional parameters: *mean branch length* and *mean summed branch length*. Mitochondrial form branching networks, in which branches intersect at a node. The mean branch length is calculated as the mean length of mitochondrial structure between two nodes. Mean summed branch length is calculated by determining the sum of branch lengths within an independent network structure and dividing by the total number of independent networks within a cell.

### 2.12. Angiogenesis-Related Protein Secretome

For the generation of HBMEC Conditioned Medium (CM), cells were plated on 6-well plates and, after infection, were maintained in a total volume of 1 mL per well. Conditioned culture media were collected at 24 h post-infection (hpi) and centrifuged for 5 min at 10,000 rpm at 4 °C and stored at −80 °C until use. Secretion of angiogenesis-related protein levels was detected using a Proteome Profiler™ Human Angiogenesis Antibody Array kit (R&D Systems) according to the manufacturer’s instructions. Membranes were incubated with pools of two independent experiments as follows: Membrane 1: Mock culture, experiment #1 + Mock culture, experiment #2; Membrane 2: MOI 0.01 experiment #1 + MOI 0.01 experiment #2; Membrane 3: MOI 0.1 experiment #1 + MOI 0.1 experiment #2). Spots were developed with chemoluminescence, and X-ray films were exposed for 1, 5, 10, and 15 min to detect differentially expressed proteins. Densitometric analysis was performed with UN-SCAN-IT gel analysis software version 7.1, and relative intensity values for each spot of the 1-min exposed film were analyzed via GraphPad Prism software version 9.0.1.

### 2.13. Transmission Electron Microscopy

Cells were grown on 35 mm Petri dishes and infected or treated as described above. At the desired time, cultures were washed in PBS, fixed with 2.5% glutaraldehyde diluted in 0.1 M cacodylate buffer with 3.5% sucrose and CaCl_2_ for 1 h at 20 °C, followed by washes in cacodylate buffer and post-fixation with 1% osmium tetroxide with potassium ferricyanide for one hour at 4 °C in the dark. Cells were dehydrated in a crescent acetone gradient and embedded in Epon resin at 60 °C for 72 h. Ultrathin sections were obtained with Leica ultramicrotome and collected in 300-mesh copper grids, stained with uranyl acetate and lead citrate, and visualized at Hitachi Transmission Electron Microscope at Centro Nacional de Bioimagem (CENABIO-UFRJ).

### 2.14. Statistical Analyses

For RT-qPCR and western blotting, a minimum of 5 independent cell culture preparations were used and analyzed with Two-Way ANOVA with Bonferroni post-test in GraphPad Prism Software v 9.3.1. Morphometrical analysis of ZO-1 immunostaining was performed with ImageJ software for fluorescence intensity and Tight Junction Organization Rate (TiJOR) using the TiJOR macro for ImageJ, which is an index of localization of tight junction proteins in the membrane–membrane contact region of adjacent cells as described by [51].

## 3. Results

### 3.1. Characterization of HBMEC Challenge by SARS-CoV-2

**Figure 1:**
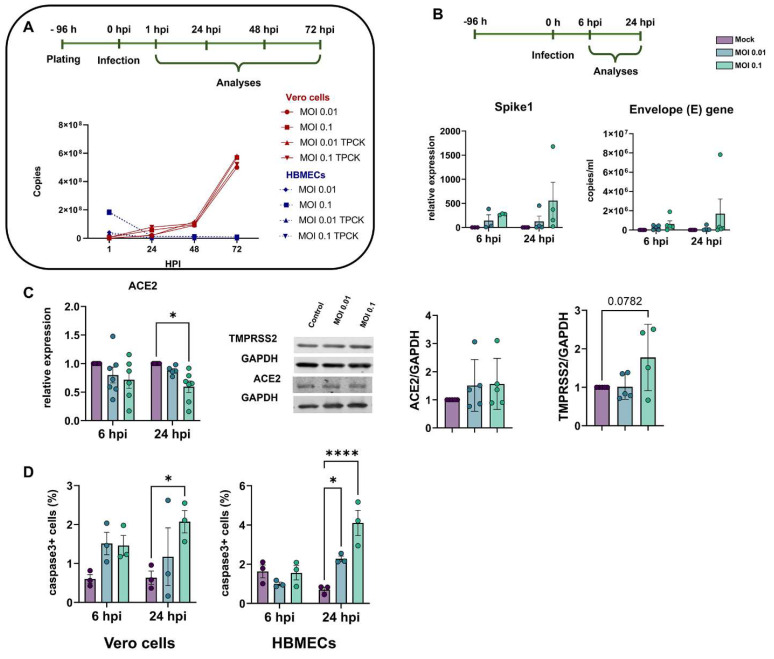
Characterization of infectivity profile of HBMECs and Vero cells by SARS-CoV-2. (**A**) Cells were exposed to different MOIs of SARS-CoV-2 (variant D614G) and viral production, and release to supernatant was analyzed by RT-qPCR for Envelope (E) gene from 0 to 72 h post-infection (hpi). As compared to Vero cells, HBMECs showed a non-productive infection. (**B**) At desired time points (6 and 24 hpi), total RNA from HBMEC cultures and expression of Spike1 and E genes were analyzed by RT-qPCR. HBMECs exposed to MOI 0.1 showed an increase in the expression of these two transcripts at 24 hpi (*p* > 0.05). (**C**) Evaluation of SARS-CoV-2 receptors expression in HBMECs after SARS-CoV-2 challenge. ACE2 mRNA had a significant decrease at 24 hpi with the MOI 0.1, which did not translate to protein levels (right panel). TMPRSS2 had a slight increase in protein content at 24 hpi. (**D**) Exposure to SARS-CoV-2 increased immunoreactivity for cleaved caspase-3 in Vero cells and HBMECs at 24 hpi. *: *p* < 0.05; ****: *p* < 0.0001, Two-Way ANOVA with Bonferroni post-test of at least five independent experiments.

In order to characterize the profile of host cell infectivity by SARS-CoV-2, HBMEC and Vero E6 cells were challenged with viral particles in the presence or not of serine endoprotease TPCK trypsin (1 μg/mL), which was shown to increase infectivity in Calu-3 cells, a permissive cell line for the efficient replication of SARS-CoV-2 [52]. Cultures were exposed to different multiplicities of infection (MOIs) of 0.01, 0.1, 1, and 2, and supernatants were collected after 6, 24, 48, and 72 h and analyzed by RT-qPCR for quantification of viral E gene (Figure 1A). We found that HBMECs showed no increase in viral replication or release in the supernatant over time, whereas Vero E6 cells had a time-dependent release of SARS-CoV-2 in the supernatant, as expected (Figure 1A). TPCK trypsin treatment did not affect the cell infectivity rates; however, for consistency, all subsequent assays were performed in the presence of TPCK. In the same context, HBMECs challenged with different MOIs did not show any increase in the expression of SARS-CoV-2 Spike1 and E genes after 6 and 24 h (Figure 1B). We showed recently that HBMEC cells express, to some extent, several SARS-CoV-2 receptors at both RNA and protein levels [8]. Therefore, we evaluated the possible effect of SARS-CoV-2 exposure on the expression of ACE2 and TMPRSS2 in HBMECs and found that exposure to MOI 0.1 induced a 40% decrease in ACE2 mRNA expression (*p* < 0.05), which did not result in ACE2 protein level alterations (Figure 1C). However, TMPRSS2 showed a 1.77-fold increase in protein levels in MOI 0.1-infected cultures at 24 hpi (*p* = 0.0782). Despite the apparent non-productive infection of HBMEC, a challenge with SARS-CoV-2 for 24 h was able to increase immunoreactivity to cleaved caspase-3, an executioner of apoptosis (Figure 1D). MOIs 0.01 and 0.1 resulted in 2.27 and 4.1% of caspase-3-positive cells, respectively, whereas non-infected dishes showed a physiological rate of 0.7% of stained cells. The apoptotic stimulus was also observed in Vero E6 cells 24 h after the challenge with MOI 0.1 (Figure 1D). Positive control with 0.5 and 2.0 µg Staurosporine for 2 h led to 1.9 and 9% of caspase-3 positive HBMECs, respectively (not shown).

### 3.2. SARS-CoV-2 Affects Tight Junction Genes Expression in BBB-Forming Cells

**Figure 2:**
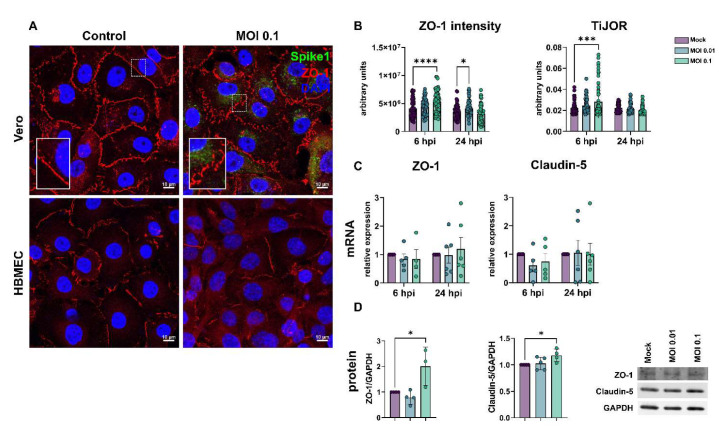
Effects of SARS-CoV-2 on tight junctional proteins in Vero and HBMECs. (**A**)**:** Cells were stained for tight junction adaptor protein ZO-1 (red) and SARS-CoV-2 Spike1 (in green). ZO-1 was affected in infected cultures at 24 hpi, as shown in higher magnification in the insets. (**B**): Morphometrical analyses of ZO-1 fluorescence intensity and TiJOR in HBMECs (**B**) showed increased ZO-1 signal and TiJOR index 6 h after exposure to the MOI 0.01. Levels of mRNA encoding for ZO-1 and claudin-5 TJ genes remained unaffected by SARS-CoV-2 challenge (**C**), but a significant increase 6 h after exposure to MOI 0.1 was observed at the protein level (**D**). *: *p* < 0.05 one-way ANOVA with Bonferroni post-test (in (**D**)) or two-way ANOVA with Bonferroni post-test (in (**B**,**C**)); ***: *p* < 0.001; ****: *p* < 0.0001, Two-Way ANOVA with Bonferroni post-test. Each symbol in (**C**,**D**) corresponds to independent cultures, and in (**B**) corresponds to microscopic field from four independent cultures. Representative blots in (**D**) from 3–4 independent experiments.

The barrier property of BMECs is mostly conferred by the expression and function of tight junction proteins, such as ZO-1 and claudin-5 [53]. HBMEC and Vero E6 cells were infected as described above and analyzed at 6 and 24 hpi. ZO-1 immunoreactivity was drastically altered in infected Vero cultures (Figure 2A) and showed discontinuous staining in cell-cell contacts, as compared to uninfected controls. SARS-CoV-2 viral particles were clearly detected in Vero cells, as revealed by Spike1 immunoreactivity. Conversely, infected HBMEC cultures did not present significant differences in the distribution of ZO-1 along cell membranes at 24 hpi (Figure 2A). To better evaluate ZO-1 organization in TJ, we performed densitometric (ZO-1 fluorescence intensity) and tight junction organization rate (TiJOR) [51] analyses in SARS-CoV-2-exposed HBMECs. We observed that ZO-1 presented a significant 1.29-fold increase in TiJOR index with exposure to MOI 0.1 at 6 hpi, concomitantly with a 1.3-fold increase in fluorescence signal, and such effects were lost after 24 h. In parallel, MOI 0.01 affected the ZO-1 fluorescence signal in HBMECs after 24 h of exposure by 1.19-fold (Figure 2B). ZO-1 and claudin-5 mRNA expression remained unaltered in HBMECs after 6 and 24 h of the SARS-CoV-2 challenge (Figure 2C), but their protein levels were significantly increased by 2.0- and 1.17-fold by the MOI 0.1 at 24 h, respectively (Figure 2D).

### 3.3. Exposure to SARS-CoV-2 Promotes Endothelial Activation and Hyper-Inflammatory Response In Vitro

**Figure 3:**
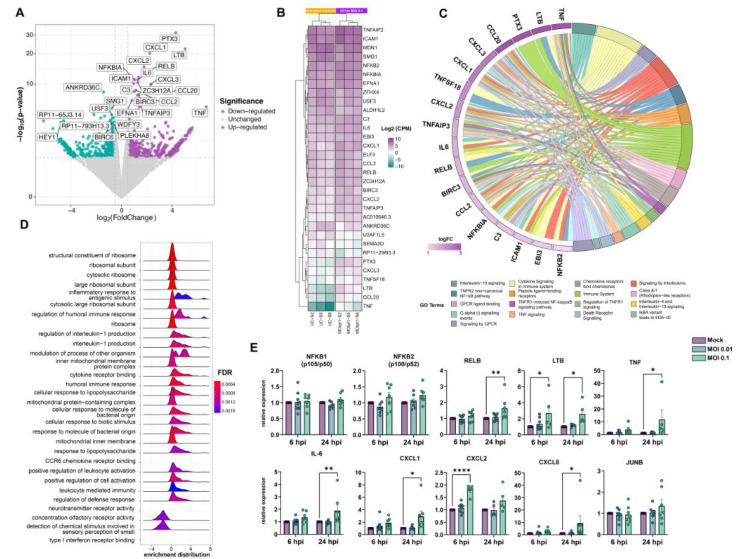
Transcriptomic profiling of SARS-CoV-2 challenge on HBMECs. Cells were exposed to MOIs 0.01 and 0.1 and analyzed by RNA-Seq. (**A**) Volcano plot depicting the overall profile of differentially expressed genes in cultures after 24 h exposure to the MOI 0.1, with up-regulated genes shown in purple and down-regulated—in green. (**B**) Heatmap diagram depicting expression levels of the most significantly altered genes by MOI 0.1 (3 right columns), as compared to uninfected controls (3 left columns). (**C**) Cnetplot visualization of functional enrichment results with up-regulated genes, depicting the functional correlation of genes with the most significant GO terms. (**D**) Enrichment functional analysis of GO terms most affected by SARS-CoV-2 challenge in HBMECs indicates inflammatory endothelial activation, as well as mitochondrial dysfunction and ribosomal-related gene expression. (**E**) RT-qPCR validation of most significantly altered genes detected in the RNA-Seq indicates activation of non-canonical NF-κB pathway, with massive increase in TNF-α, lymphotoxin B (LTB, or TNF-C), and downstream target genes, such as IL-6, CXCL1, -2, and -8. NFKB1 (p105/p50) and NFKB2 (p100/p52), as well as JUNB, showed no significant alteration in SARS-CoV-2-exposed cultures. *: *p* < 0.05; **: *p* < 0.01; ****: *p* < 0.0001, two-way ANOVA with Bonferroni post-test of at least 5 independent experiments. MOI: multiplicity of infection; GO: gene ontology.

**Figure 4:**
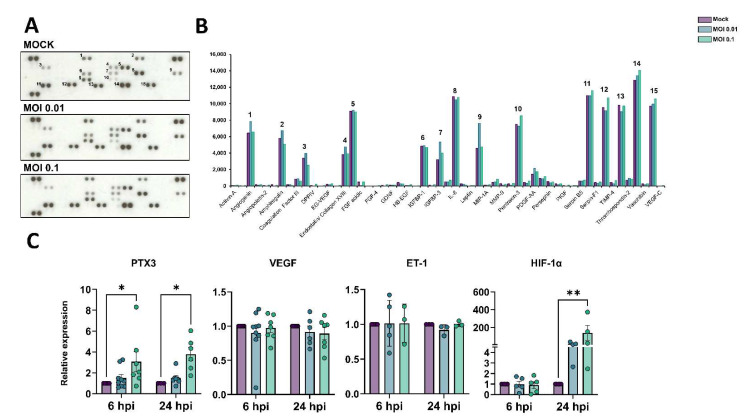
Production of angiogenic-related molecules is modulated by SARS-CoV-2 in HBMECs. (**A**) Conditioned medium from Mock and SARS-CoV-2-exposed HBMEC cultures (both with MOI 0.01 and 0.1) were analyzed via Proteome Profiler Human Angiogenic Antibody Array and detected by chemoluminescence, each protein detected in duplicated spots. (**B**) Densitometric analysis of membranes in (**A**) revealed the analytes with the strongest signal and which were affected by the SARS-CoV-2 challenge. Spots labelled 1-15 in (**A**) correspond to the analytes depicted in (**B**). (**C**) RT-qPCR analysis of angiogenesis-related genes in HBMECs revealed that PTX3 and HIF-1α were increased following SARS-CoV-2 exposure. *: *p* < 0.05; **: *p* < 0.01, two-way ANOVA with Bonferroni post-test of at least five independent experiments.

We performed RNA-Seq analyses 6 and 24 h after exposing HBMECs to SARS-CoV-2 to determine their transcriptional profiles. At 6 h, biological replicas had high variability across experiments, as determined by the square root of the common dispersion and visualized by principal component analysis (not shown). Exposure to both MOIs 0.01 and 0.1 led to minimal effect on HBMEC’s transcriptome, with few significantly differentially expressed genes (DEGs) and no pathways enrichment found (Supplementary Material). However, at 24 h, we observed a significant impact on host cell transcriptome: exposure to SARS-CoV-2 MOI 0.1 led to the up-regulation of 23 and down-regulation of four genes. The volcano plot and heatmap in Figure 3A,B, respectively, depict the transcriptomic profile of HBMECs exposed to the MOI 0.1 at 24 hpi. Data obtained from RNA-Seq was consistent with endothelial activation, with high expression levels of cytokines (IL-6, IL-8, TNF) and chemokine (CXCL1, -2, -8, and CCL20) encoding genes (Figure 3). Accordingly, functional enrichment analysis revealed that the main genes found related to “Cytokine signaling in the immune system”, “TNF signaling”, and “TNFR1-induced NFkappaB signaling pathway”, among other Reactome terms (Figure 3C). In fact, TNF was the most up-regulated gene, with a 104-fold increase, followed by TNF-c (Lymphotoxin beta, LTB), with a 32.8-fold change (Figure 3, Table 1). Interestingly, LTB is a known inducer of the noncanonical NFκB inflammatory pathway [54] and was found to be up-regulated both at 6 and 24 h in SARS-CoV-2-exposed HBMEC by RT-qPCR (Figure 3E). Although our RNA-Seq data revealed an increase in NFκB2 (p100/p52) and NFκBIA (IκBα), we performed RT-qPCRs with additional biological samples for NF-κB1 (p105/p50) and NF-κB2 and observed that due to biological variability such genes remained unaltered in challenged cultures (Figure 3D). However, RELB, the main activator of the noncanonical NF-κB signaling pathway [54], was shown to be up-regulated by confirmatory RT-qPCR at 24 h (Figure 4). We also further confirmed by RT-qPCR the up-regulation of inflammation-related genes, including LTB, TNF, IL-6, CXCL1, CXCL2, and CXCL8 (Figure 3E). Pentraxin3 (PTX3) is a glycoprotein involved in the innate immune response and has a relevant role in FGF2-dependent angiogenesis [55]. We found PTX3 to be 19.6-fold-increased in SARS-CoV-2-exposed HBMECs (Figure 3A and Figure 4). Apart from the inflammatory transcriptomic response, KEGG pathways related to ribosomal structure/function and mitochondrial biology were found to be altered by SARS-CoV-2-exposed HBMECs at 24 h (Figure 3D).

### 3.4. Angiogenic Profiling of SARS-CoV-2-Exposed HBMEC Cells

Dysfunctional angiogenesis is a common phenomenon observed in neuroinflammatory states and can be a result of BBB damage [56]. We analyzed the profile of angiogenesis-related secreted proteins by HBMECs during the challenge with SARS-CoV-2 and observed that out of 55 spotted targets, 15 had the most significantly detectable signals (Figure 3A,B, Table 2). The highest signals were observed for uPA, serpin-E1 (PAI-1), IL-8, thrombospondin-1, VEGF, TIMP-1, endothelin-1 (ET-1), PTX3, angiogenin, and amphiregulin, with at least 5000 pixels each. SARS-CoV-2-exposed cultures (MOI 0.1) had the most pronounced increase in the secretion of PTX-3 and TIMP-1 as compared to Mock-treated cultures, with 113 and 112% levels, respectively. Accordingly, PTX-3 was also one of the most up-regulated genes as determined by RNA-Seq (Figure 3). We further assessed the expression levels of PTX3 by RT-qPCR and found it to be increased in HBMEC cultures after 6 and 24 h exposure with the MOI 0.1, whereas VEGF and ET-1 showed no significant changes at the transcriptional level (Figure 4C). Insulin-like growth factor binding protein-3 (IGFBP-3), a member of the IGFBP family, was shown to be 166 and 125% more abundant in the HBMEC-conditioned media in MOI 0.01 and 0.1-treated dishes, respectively. Interestingly, monocyte chemoattractant protein 1 (MCP-1) had a selective increase in secreted levels in HBMEC cultures exposed to MOI 0.01, shifting from 4549 pixels in Mock-treated cultures to 7624 pixels in MOI 0.01-exposed cultures, which corresponds to a 1.67-fold increase; whereas MOI 0.1-exposed cultures showed a 4759-pixel signal for MCP-1. We performed scratch-wound healing migration assays in infected HBMECs; however, no effect in cellular migration was noticed in challenged cultures compared to Mock-treated cultures (not shown). Interestingly, hypoxia-inducible factor-1 alpha (HIF-1α) was also increased by SARS-CoV-2 challenge at both MOIs at 24 hpi (Figure 4C).

### 3.5. Mitochondrial Plasticity Is Affected by Exposure to SARS-CoV-2

**Figure 5:**
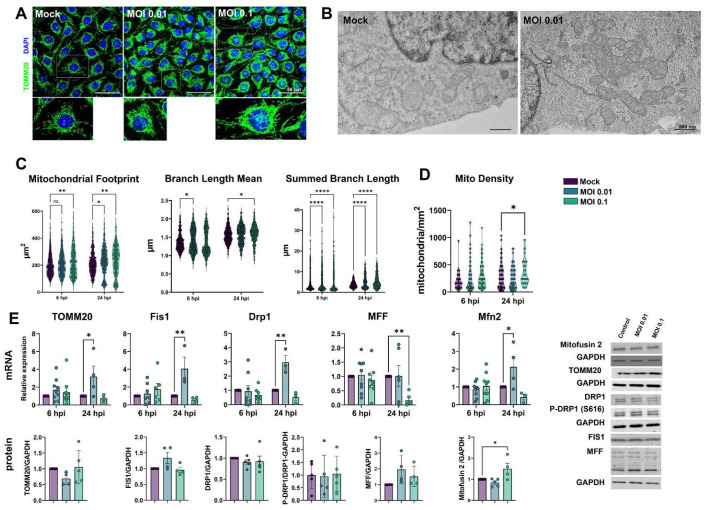
SARS-CoV-2-induced mitochondrial remodeling in HBMECs. Mitochondrial networks were detected by TOMM20 immunostaining (**A**) and TEM (**B**). MiNA analysis of TOMM20 revealed that exposure to SARS-CoV-2 induced an increase in mitochondrial footprint, branch length mean, and summed branch length (**C**). Mitochondrial density was calculated by TEM images (**D**), which also revealed increased fusion and association with multivesicular bodies (**B**). (**E**): RT-qPCR (**upper panel**) and western blotting (**lower panel**) analyses revealed that although fission-related genes (Fis1 and Drp1) were up-regulated in MOI 0.01-exposed cultures, only Mfn2 protein levels were increased in MOI 0.1-exposed cultures. TOMM20 protein levels also remained unaltered. *: *p* < 0.05; **: *p* < 0.01; ****: *p* < 0.0001, two-way ANOVA with Bonferroni post-test. Each symbol in graphs represents one cell (**C**), one mitochondrion (**D**), or one independent experiment (**E**). Bottom right panels depict blots from (**E**). Scale bars: 50 µm for (**A**) and 500 nm for (**B**).

Because mitochondria play a role in cellular homeostasis and pathology, we sought to investigate the effects of the SARS-CoV-2 challenge on mitochondrial plasticity in HBMECs. Cells were immunostained for mitochondrial import receptor subunit TOMM20 (Figure 5A). Our first observation was that MOIs 0.01 and 0.1 induced a denser mitochondrial network profile when compared to the Mock-treated condition (Figure 5A–C). Mitochondrial Network Analysis (MiNA) [47] revealed that *mitochondrial footprint* (Figure 5C), which measures the mitochondria signal in a 2-dimensional image of a cell, was found to be significantly increased in HBMECs challenged with the MOI 0.1 at 6 h and with both MOIs at 24 h. We next measured the mean mitochondrial *branch length mean*, which is the average length of mitochondrial structures that are either independent or connected to networks (Figure 5C). We observed a slight, yet significant, increase in cells exposed to MOI 0.01 at 6 h and with MOI 0.1 at 24 h, with a 7 and 3% increase, respectively. Furthermore, MiNA revealed that SARS-CoV-2 induced an overall increase in mitochondrial networks, with a significant increase in *summed branch length mean* values at 6 (34 and 33% increase for MOIs 0.01 and 0.1, respectively) and 24 hpi (38 and 45% increase for MOIs 0.01 and 0.1, respectively). Mitochondrial morphological analyses were further assessed by TEM (Figure 5B), and we found that challenged HBMECs displayed larger, swollen mitochondria with reduced cristae and, to some extent, associated with multivesicular bodies. Moreover, MOI 0.1-treated cultures displayed 356 mitochondria/mm^2^, while Mock-infected cultures had 266 mitochondria/mm^2^ (*p* < 0.05), which corresponded to a 33% increase (Figure 5D).

Since changes in mitochondrial networks could be influenced by abnormal fission or fusion events [57], we evaluated the expression of markers of such processes. TOMM20, used to determine mitochondrial networks by confocal microscopy and MiNA analysis (Figure 5A,C), had a four-fold increase in its mRNA level (*p* > 0.05) in MOI 0.01-exposed cells. However, no changes were detected in TOMM20 protein levels by western blotting (Figure 5E). We then assessed the expression of mitochondrial fission-related genes. Fis1 and Drp1 mRNA were significantly increased by 4-fold and 3-fold, respectively, at 24 hpi in HBMECs exposed to the MOI 0.01, which did not translate to changes in Fis1 and Drp1 protein content (Figure 5E). Drp1 phosphorylation at serine 616 (Drpi1^S616^) residue, which is responsible for directing mitochondrial fission [58], was not altered in SARS-CoV-2-exposed cultures. Mitochondrial Fission Factor (MFF) showed a 0.84-fold reduction induced by MOI 0.1 at 24 hpi, which also did not reflect in altered protein expression. However, Mitofusin-2 (Mfn2) mRNA levels showed a 4-fold increase by MOI 0.01 at 24 h (*p* = 0.02), while Mfn2 protein levels were increased by 1.5-fold in MOI 0.1-exposed cultures as compared to Mock-treated (Figure 5E). 

## 4. Discussion

Neurological consequences of COVID-19 still pose a relevant puzzle to the medical and scientific community. Since its first cases, CNS invasion has been described [59,60], but the routes and mechanisms by which SARS-CoV-2 gains entry to brain parenchyma remain elusive [61]. In the present study, we aimed to deepen previous observations of our group that exposure to SARS-CoV-2 proteins led to HBMEC cellular responses, such as tight junction protein remodeling [8]. Despite our previous observation that primary HBMECs express several receptors for the virus [8], we found little to no indication of productive viral replication in the supernatants of HBMECs, which is in accordance with previous reports that described that several endothelial cell types are not permissive for SARS-CoV-2 productive infection [62,63]. Krasemann et al. [64] observed infection of iPS-derived HBMECs but only at MOIs 10 and 100, which are unlikely to have pathological significance.

Exposure to SARS-CoV-2 did not affect the expression of ACE2 and TMPRSS2, and, despite the apparent lack of productive infection, exposure to SARS-CoV-2 increased cleaved caspase-3 immunoreactivity, an indicator of apoptotic cell death. This effect was observed both in HBMECs and Vero epithelial cells and is consistent with what was described in the literature [65]. In fact, HBMECs have been shown to undergo cell death in response to viral infections, including Dengue [66] and Zika [67,68] viruses, followed or not by changes in BBB permeability. These results suggest that the interaction of host cells with viral surface proteins may be sufficient to trigger programmed cell death cascades even in the absence of a productive infection.

Tight junction proteins have a crucial role in maintaining BBB integrity and its selective paracellular permeability [53]. In our study, SARS-CoV-2-infected Vero E6 cells showed marked disorganization of paracellular tight junctions, as shown by ZO-1 immunostaining. Previous studies described that treatment of HBMECs in 2D or 3D cultures with the S1 subunit Spike1 protein led to mislocalization of ZO-1, concomitantly with cytokine secretion [33,69]. Not only ZO-1 [70] but also β-catenin, cadherin-5ccludingcludin junctional proteins have recently been shown to be affected by SARS-CoV-2 proteins in HUVECs [71]. ZO-1 possesses a PDZ domain, which is responsible for binding and interacting with other proteins [72,73]. Interestingly, ACE2 possesses a PDZ-binding domain [74,75], and it has been suggested that epitopes of viral proteins, such as 1–60: M1Lys60 and 241–300: Ala240-Glu300 could directly bind to ZO-1 and VCAM-1 PDZ domains, thus suggesting a possible alternative route of CNS entry. We found ZO-1 and claudin-5 protein levels to be increased after 24 h of SARS-CoV-2 exposure in HBMECs, and this increase is consistent with what our group recently demonstrated with S1 treatment [8]. Whether ZO-1 increase can be directly related to BBB permeability may vary among experimental models and infectious agents. We have described that the Honduras isolate of Zika virus selectively up-regulated ZO-1 expression in vitro, while BBB permeability was increased in vivo [67]. In fact, proper BBB functioning relies on the combined expression, phosphorylation, and/or localization of ZO-1, -2, and -3, claudin-5, occludins, and tricellulin [76,77]. Direct infection with higher MOIs of SARS-CoV-2 or treatment with plasma from COVID-19 patients failed to induce significant increases in permeability in BMECs in vitro [78]. Conversely, using the K18 mouse model and hamster infection, Zhang et al. [79] showed that SARS-CoV-2 effectively infects and replicates in HBMECs, but leads to no change in BBB permeability and TJ proteins. Interestingly, a massive inoculation of iPS-derived HBMECs (MOIs 10 and 100) showed active viral replication, whereas MOIs 0.1 and 1 described infectivity near 0.6% cells, which is similar to what we observed herein [64].

Following the initial characterization of the cellular effects of HBMECs after the SARS-CoV-2 challenge, we sought to characterize the transcriptomic landscape of BBB-forming cells after such treatment. Previous data from our group have demonstrated that primary HBMECs exposed to the S1 subunit of SARS-CoV-2 Spike1 protein led to alterations in tight junction gene/protein expression [8]. Therefore, our aim was to deepen the knowledge of what molecular pathways are affected by SARS-CoV-2 proteins. We performed RNA-Seq analyses of HBMECs after 6 and 24 h challenges with SARS-CoV-2 MOIs 0.1 and 0.01. Due to the low change in the overall host cell transcriptome with most of the experimental conditions used in this study, we focused our subsequent analyses of the MOI 0.1 treatment at 24 hpi. The majority of the significantly up-regulated genes corresponded to known endothelial activation pathways, including CXCL1, -2, -3, CCL20, PTX3, ICAM1, and TNF. Combined upregulation of LTB, TNF, and RELB by SARS-CoV-2 provided evidence that activation of the non-canonical NF-κB pathway activation may be taking place. NF-κB is a family of transcription factors that can be activated by several ligands and activates the expression of proinflammatory cytokines and chemokines [80]. Interestingly, the main protease of SARS-CoV-2 (M^pr^°) cleaves a member of the NF-κB family, NEMO, which, in turn, leads to HBMEC cell death in vitro and in vivo [81]. RELB can form heterodimers with p50/p105, p52/p100, and p65 [54,82] but can also bind to sirtuin1 to direct epigenetic silencing of inflammatory gene expression [83,84]. In fact, among KEGG pathways enriched in our datasets, we found ribosomal structure and function as possible candidates for epigenetic regulation induced by SARS-CoV-2. These observations suggest that host epigenetic factors may be key for the outcome of COVID-19 and/or long COVID-19. Indeed, the promoters of the genes involved in inflammation, including NF-κB, can be demethylated, thereby resulting in an increased expression of interferons (IFNs), possibly leading to a “cytokine storm” [85]. The expression of IL-6, another important player in the so-called cytokine storm occurring in the most severe COVID-19 patients, was also significantly increased in infected HBMEC, and it is known to be modulated by its promoter methylation. It was also observed that oxidative stress induced by viral infections, including SARS-CoV-2 infection, can inhibit the maintenance of DNA methyltransferase-1 (DNMT1), thereby aggravating the DNA methylation defects [86,87,88]. Our preliminary results (not shown) indicate that HBMEC exposure to SARS-CoV-2 results in a decrease in DNA methylation, supporting a recent study of genome-wide DNA methylation analysis in peripheral blood of COVID-19-infected individuals, which identified marked epigenetic signatures, such as hypermethylation of IFN-related genes and hypomethylation of inflammatory genes [89]. Such observations further suggest the involvement of epigenetic regulatory mechanisms in COVID-19 [90]. MCP-1, a pro-inflammatory chemokine well-known to be increased in COVID-19 patients [91,92,93,94,95], was also found to be increased in SARS-CoV-2-exposed HBMEC conditioned media, as detected by mini-proteome assays. Accordingly, recent findings from our group show that both delta and D614G Spike1 proteins are capable of inducing MCP-1 release from HBMECs (Stangis and Toborek et al., unpublished data). MCP-1 plays a key role in leukocyte migration to the brain parenchyma [96] and can be used as a biomarker of HIV-1-induced neuroinflammation/neuropathogenesis [97,98].

As stated above, PTX3 was one of the main hits found in the transcriptomic analyses. Pentraxins are a superfamily of multifunctional proteins with conserved phylogeny [99], divided into two groups based on their primary structure: short and long pentraxin, where c-reactive protein and PTX are examples of short and long pentraxins, respectively. PTX3 is expressed in several neural cell types [100,101,102,103,104] and in endothelial cells and can be upregulated by inflammatory stress, such as cytokine stimulation [105]. We performed profiling of angiogenesis-related panels in the supernatants of SARS-CoV-2-challenged HBMEC and confirmed that PTX3 was increased by this treatment. PTX3 is known to be produced in high amounts by blood vessels in vascular inflammatory conditions [106] and inhibits FGF2-dependent angiogenesis [55,107]. Pathological vascularization and angiogenesis have been described as a unique comorbidity associated with SARS-CoV-2 infection in the pulmonary endothelium [108], including microvascular distortion and increased intussusceptive angiogenesis [109,110]. Moreover, VEGF, as well as other angiogenic-related analytes, were found to be increased in COVID-19 patients (including PTX3), which correlated with disease severity [111]. Accordingly, VEGF levels were 8% increased in the supernatants of SARS-CoV-2-exposed HBMECs, even though *vegf* transcripts remained unaltered. It is well-known that inflammation, especially IL-6-dependent, can stimulate defective angiogenesis [112], and our data further contributes to the notion that following SARS-CoV-2 infection, there is an intense brain endothelial activation, which leads to defective angiogenic signaling and possibly endothelial permeability. Additionally, we found HIF-1α to be greatly increased after 24 h of treatment. HIF-1α is a major angiogenesis inductor and is known to be up-regulated by distinct viral infections (reviewed by [113]). HIF-1α is activated and translocated to the nucleus upon hypoxic conditions [114], and it has been shown that COVID-19 patients present massive hypoxia due to vasoconstriction and coagulopathy [115]. Interestingly, ACE2 expression is decreased in pulmonary smooth muscle cells upon HIF-1α accumulation [116], whereas hypoxia leads to a biphasic modulation of both ACE2 and TMPRSS2 expression on brain microvascular endothelial cells (hCMEC/D3), with an initial increase at 6 h and a decrease at 48 h of hypoxic stimulus cells [117]. These observations are in accordance with our present data, that ACE2 is decreased while HIF-1α is increased at 24 hpi. Although VEGF is one of the most described downstream targets of HIF-1α activation, apoptotic cell death, and IFN-stimulated gene expression are additional targets of HIF-1α activation [113], which can also be dependent on NF-κB signaling pathway [118]. Our data indicate that HIF-1α up-regulation can be a part of a SARS-CoV-2-induced endothelial activation, along with cytokine/chemokine stimulation and NF-κB non-canonical activation.

Our final series of experiments focused on mitochondrial morphology and dynamics in HBMECs following SARS-CoV-2 exposure. It is well known that mitochondria are gatekeepers of BBB endothelium physiology and correspond to higher cytoplasmic volume as compared to non-cerebrovascular endothelial cells [119,120]. Moreover, mitochondrial function is important for BBB maintenance and integrity [121]. It was described that SARS-CoV encodes a protein named open reading frame-9b (ORF-9b), which localizes to mitochondria, increases Drp1-mediated mitochondrial elongation, and activates innate cellular response via MAVS signalosome [122]. We first employed a morphological/morphometrical approach to determine the mitochondrial contents and cellular distribution. Herein we demonstrated that direct exposure to SARS-CoV-2 led to a remodeling of mitochondrial networks. By using the MiNA plugin, we verified that challenged HBMECs had increased mitochondrial footprint as an estimation of the overall TOMM20 pixel signal. Recent reports have also shown an effect of SARS-CoV-2 and COVID-19 on mitochondrial biology: monocytes isolated from COVID-19 patients display reduced mitochondrial membrane potential, and SARS-CoV-2 viral load was positively correlated with the generation of ROS [123]. Importantly, endothelial cells exposed to SARS-CoV-2 Spike1 protein showed decreased tubular and increased fragmented mitochondrial networks in vitro, which was accompanied by a decrease in oxygen consumption rate and increased extracellular acidification rate [124]. Confirming observation was recently described by Domizio et al. [125], in which pulmonary endothelial cells in a lung-on-a-chip infection model displayed increased mitochondrial networks. Similarly, we demonstrated that mitochondrial networks were increased, as determined by summed branch length analyses, which indicates that SARS-CoV-2-exposed HBMECs had longer mitochondrial ramifications. Changes in endothelial mito-morphology are well described in several models of inflammatory diseases and/or aging [126,127,128] and are correlated with abnormalities in the mitochondrial quality control system, which in turn, can lead to increased ROS production. Mitochondrial quality control encompasses biogenesis, fission, fusion, and mitophagy processes, which are essential for its biology and function. We analyzed mitochondrial fusion and fission markers after exposing HBMECs to different concentrations of SARS-CoV-2. Our results showed a significant increase in the fission and fusion-related gene expression Fis1, Drp1, and Mfn2 and a trend to increase MFF when cells were exposed to MOI 0.01. Interestingly, this effect is opposite when cells are exposed to MOI 0.1, showing a significant decrease in the MFF mRNA levels as well as a trend to decrease Fis1, Drp1, and Mfn2, possibly as a compensatory mechanism. However, Mfn2 was the only mitochondrial plasticity marker, which showed a significant increase in protein levels in HBMECs exposed to MOI 0.1, which could explain the increased values in morphometric MiNA data that revealed increased branch length means. Confirming our results, several studies had shown the relationship between endothelial cell dysfunction and mitochondria fusion and fission balance in response to cellular damage [128,129,130,131]. Moreover, we found mitochondria associated to some extent with multivesicular bodies, which has also been described as another pathway for mitochondrial quality control [132,133]. Interestingly, several reports have linked NF-κB-mediated inflammation with mitochondrial responses [134,135,136,137], which could indicate that, in fact, mitochondrial remodeling observed in infected HBMECs could be due to (or lead to) SARS-CoV-2-induced inflammatory response.

## 5. Conclusions

Despite no active replication or signs of productive infection, exposure to SARS-CoV-2 leads brain microvascular endothelial cells to a proinflammatory activation, possibly mediated by NF-κB non-canonical pathway activation. These events would result in mitochondrial and tight junction remodeling and endothelial apoptosis. Taken together, our data point to a relevant role of circulating SARS-CoV-2 viral particles or proteins on BBB-forming endothelial cells, which could contribute to a neuroinflammatory state. These events reflect important aspects of clinical observations of neurological and cerebral vascular manifestations of COVID-19.

## Figures and Tables

**Table 1:** Differentially expressed genes after 24 h of HBMEC exposure to MOI 0.1.

Gene Name	Symbol	Fold-Change (Log)	*p* Value	FDR
Uncategorized gene	RP11-298I3.3	−2.19	0.00001795234598	0.04214061886
ankyrin repeat domain 36C	ANKRD36C	−1.42	0.000000005528613831	0.00002495701339
upstream transcription factor family member 3	USF3	−0.76	0.00002036226065	0.04595918861
SMG1 nonsense mediated mRNA decay associated PI3K related kinase	SMG1	−0.75	0.000002651386726	0.007779698933
nuclear factor kappa B subunit 2	NFKB2	0.64	0.00002117775188	0.04602945153
E74 like ETS transcription factor 3	ELF3	0.82	0.00001027948854	0.02601045479
ephrin A1	EFNA1	0.82	0.0000001766175517	0.0006096837885
Epstein–Barr-virus-induced 3	EBI3	0.87	0.000005771957935	0.01612959902
intercellular adhesion molecule 1	ICAM1	0.97	0	0.0000005593411357
complement C3	C3	1.01	0.000000004506547278	0.00002203851837
NFKB inhibitor alpha	NFKBIA	1.05	0	0.00000008288304584
C-C motif chemokine ligand 2	CCL2	1.14	0.000000002315586268	0.00001235344223
baculoviral IAP repeat containing 3	BIRC3	1.27	0.00000002052506134	0.00007528079373
zinc finger CCCH-type containing 12A	ZC3H12A	1.29	0	0.0000003145723235
RELB proto-oncogene, NF-kB subunit	RELB	1.30	0	0.00000008288304584
Uncharacterized protein	AC010646.3	1.31	0.0000103901062	0.02601045479
interleukin 6	IL6	1.36	0	0.00000002581261588
TNF alpha induced protein 3	TNFAIP3	1.38	0.00000001761165902	0.00006890150651
TNF alpha induced protein 2	TNFAIP2	1.42	0.000001921435199	0.005934605433
C-X-C motif chemokine ligand 2	CXCL2	1.79	0	0
TNF superfamily member 18	TNFSF18	1.95	0.0000106374977	0.02601045479
C-X-C motif chemokine ligand 1	CXCL1	2.24	0	0
C-X-C motif chemokine ligand 3	CXCL3	2.32	0.0000000001312489102	0.0000007702211048
C-C motif chemokine ligand 20	CCL20	3.50	0.000000007991169552	0.00003349669957
pentraxin 3	PTX3	4.30	0	0
lymphotoxin beta	LTB	5.04	0	0

**Table 2:** Differentially secreted angiogenic proteins in SARS-CoV-2-exposed HBMECs.

Analyte Name (Alternate Nomenclature)	Gene ID	Foreground Pixel Intensity (Fold Change Relative to Mock)		
		Mock	MOI 0.01	MOI 0.1
**Activin-A**	3624	55.5	32.5 (0.6)	88.5 (1.63)
ADAMTS-1	9510	25.5	12.5 (0.52)	45.5 (1.88)
**Angiogenin (ANG)**	283	6427	7818 (1.21)	6553 (1.01)
Angiopoietin-1 (Ang-1)	284	147	86 (0.59)	155 (1.07)
Angiopoietin-2 (Ang-2)	285	39.5	12 (0.32)	79.5 (2.08)
Angiostatin/ Plasminigen	5340	144.5	10 (0.07)	74 (0.52)
**Amphiregulin (AR)**	374	5797	6744 (1.16)	5064.5 (0.87)
Artemin	9048	144.5	143 (0.99)	64 (0.46)
Coagulation Factor III (TF)	2152	818	887 (1.08)	622 (0.76)
**CXCL16**	58,191	3387.5	3976.5 (1.17)	2521.5 (0.74)
DPPIV (CD26)	1803	72.5	1.5 (0.03)	203 (2.83)
EGF	1950	16.5	18 (1.15)	40.5 (2.60)
EG-VEGF (PK1)	84,432	188.5	164 (0.88)	257 (1.38)
Endoglin (CD105)	2022	17.5	17.5 (1.05)	74.5 (4.40)
**Endostatin/ Collagen XVIII**	80,781	3827	4747.5 (1.24)	3931.5 (1.02)
**Endothelin-1 (ET-1)**	1906	9087.5	9213 (1.01)	9007.5 (0.99)
FGF acidic (FGF-1)	2246	475	69.5 (0.15)	472.5 (1)
FGF basic (FGF2)	2247	16.5	5 (0.36)	4.5 (0.42)
FGF-4	2249	0.5	−1	36.5 (78.0)
FGF-7 (KGF)	2252	77.5	15.5 (0.21)	180 (2.34)
GDNF	2668	101	82 (0.82)	105 (1.06)
GM-CSF	1437	419.5	275 (0.65)	241.5 (0.58)
HB-EGF	1839	71	32.5 (0.47)	134 (1.92)
HGF	3082	10.5	11 (1.14)	25.5 (2.66)
**IGFBP-1**	3484	4841.5	4901 (1.01)	4684.5 (0.96)
IGFBP-2	3485	118	22.5 (0.19)	154.5 (1.33)
**IGFBP-3**	3486	3203	5335.5 (1.66)	3991 (1.24)
IL-1β (IL-1F2)	3553	512	470.5 (0.92)	695.5 (1.36).
**IL-8 (CXCL8)**	3576	10,875.5	10,484.5 (0.96)	10,744 (0.98)
LAP (TGF B1)	7040	292.5	219.5 (0.75)	102 (0.35)
Leptin	3952	1	44 (45.0)	43.5 (46.0)
**MCP-1 (CCL2)**	6347	4549	7624 (1.67)	4759 (1.04)
MIP-1A (CCL3)	6348	123	103 (0.84)	149 (1.23)
MMP-8	4317	418	510.5 (1.22)	841.5 (2.01)
MMP-9	4318	298.5	106.5 (0.36)	206 (0.69)
NRG1-B1 (HRG1-β1)	3084	256	107.5 (0.42)	320 (1.25)
**Pentraxin-3 (TSG-14)**	5806	7509.5	7263.5 (0.96)	8523 (1.13)
PD-ECGF	1890	441	310 (0.70)	600 (1.36)
PDGF-AA	5154	1411.5	2129.5 (1.50)	1736.5 (1.23)
PDGF-AB/ PDGF-BB	5155	923.5	878 (0.95)	1161 (1.25)
Persephin	5623	565.5	375.5 (0.66)	490.5 (0.87)
Platelet Factor 4 (CXCL4)	5196	261.5	150.5 (0.58)	283.5 (1.09)
PIGF	5228	50.5	14 (0.29)	90 (1.83)
Prolactin	5617	7	49 (7.14)	57 (8.5)
Serpin B5 (Maspin)	5268	604	578 (0.95)	694 (1.15)
**Serpin-E1 (PAI-1)**	5054	10,979.5	10,955.5 (0.99)	11,590.5 (1.05)
Serpin-F1 (PEDF)	5176	422.5	327.5 (0.77)	476 (1.13)
**TIMP-1**	7076	9548	9156 (0.95)	10,696 (1.12)
TIMP-4	7079	446.5	292.5 (0.65)	656.5 (1.47)
**Thrombospondin-1 (TSP-1)**	7057	9842	9012 (0.91)	9727.5 (0.98)
Thrombospondin-2 (TSP-2)	7058	688	929 (1.35)	837 (1.22)
**uPA**	5328	12,865	13,396.5 (1.04)	14,065 (1.09)
Vasohibin	22,846	253	172 (0.68)	268 (1.06)
**VEGF**	7422	9700.5	9954 (10.2)	10,559.5 (1.08)
VEGF-C	7424	0.5	0	37 (79.0)

Analytes in bold had at least 3000 pixels and were considered as valid signals, as shown in Figure 4.

## Data Availability

RNA-Seq raw data will be deposited upon acceptance of this manuscript, and such information will be added to the METHODS section.

## Supplementary Materials

The following supporting information can be downloaded at: https://www.mdpi.com/article/10.3390/v15030745/s1, Table S1: RT-qPCR primer sequences used in this work; Table S2: Antibodies used in this work.

## Abbreviations

2D: two-dimensional; 3D: three-dimensional; ACE2: Angiotensin-converting enzyme 2; ADAM17: A disintegrin and metalloprotease 17; AGTR2: Angiotensin II receptor type 2 (AT_2_ receptor); ANOVA: Analysis of Variance; ANPEP: Aminopeptidase N; BBB: Blood–brain barrier; BCA: Bicinchoninic acid; BSG/CD147: Basigin/ cluster of differentiation 147; CCL20: C-C Motif Chemokine Ligand 20; cDNA: complementary DNA; CM: conditioned medium; CNS: Central Nervous System; COVID-19: Coronavirus disease 2019; CXCL1: chemokine (C-X-C motif) ligand 1; CXCL2: chemokine (C-X-C motif) ligand 2; CXCL8: chemokine (C-X-C motif) ligand 8; DNA: Deoxyribonucleic acid; DPP4: Dipeptidyl peptidase-4; E gene: envelope gene; FGF-2: fibroblast growth factor (or basic FGF); HBMEC: human brain microvascular endothelial cells; HPI: hours post-infection; HUVEC: human umbilical vein endothelial cell; IL-6: interleukin-6; IL-8: interleukin-8; iPS: Induced pluripotent stem cell; Mfn2: mitofusin-2; MOI: multiplicity of infection; NF-κB: Nuclear factor kappa-light-chain-enhancer of activated B cells; PBS: phosphate buffered saline; PTX3: Pentraxin3; RNA: Ribonucleic acid; S1: Spike1; SDS: Sodium Dodecyl sulfate; SV40-LT: Simian vacuolating virus 40- large T antigen; TBS: Tris-buffered saline; TIMP-1: tissue inhibitor of metalloproteinase-1; TMPRSS2: Transmembrane protease, serine 2; TNA-α: tumor necrosis factor- alpha; TNF-c: tumor necrosis factor-c; TOMM20: Mitochondrial import receptor subunit TOM20 homolog; TPCK: Tosyl phenylalanyl chloromethyl ketone; uPA: urokinase-type plasminogen activator; VCAM-1: Vascular cell adhesion protein 1; VEGF: vascular endothelial growth factor; WHO: World Health Organization; ZO-1: zona occludens-1

## References

[1] Centers for Disease Control and Prevention (CDC) COVID Data Tracker. http://www.cdc.gov.

[2] WHO (2021). Laboratory biosafety guidance related to coronavirus disease (COVID-19): Interim Guidance, 28 January 2021. https://www.who.int/publications/i/item/WHO-WPE-GIH-2021.1.

[3] Centers for Disease Control and Prevention Case-Surveillance. https://data.cdc.gov.

[4] Zhu N., Zhang D., Wang W., Li X., Yang B., Song J., Zhao X., Huang B., Shi W., Lu R. (2020). China Novel Coronavirus Investigating and Research Team. A novel coronavirus from patients with pneumonia in China. N. Engl. J. Med..

[5] Wang K., Chen W., Zhang Z., Deng Y., Lian J.Q., Du P., Wei D., Zhang Y., Sun X.X., Gong L. (2020). CD147-spike protein is a novel route for SARS-CoV-2 infection to host cells. Signal Transduct. Target. Ther..

[6] Coronaviridae Study Group of the International Committee on Taxonomy of Viruses (2020). The species Severe acute respiratory syndrome-related coronavirus: Classifying 2019-nCoV and naming it SARS-CoV-2. Nat. Microbiol..

[7] Ni J., Xiao S., Li X., Sun L. (2019). ACE gene missense mutation in a case with early-onset, rapid progressing dementia. Gen. Psychiatry.

[8] Torices S., Cabrera R., Stangis M., Naranjo O., Fattakhov N., Teglas T., Adesse D., Toborek M. (2021). Expression of SARS-CoV-2-related receptors in cells of the neurovascular unit: Implications for HIV-1 infection. J. Neuroinflamm..

[9] Hoffmann M., Kleine-Weber H., Schroeder S., Krüger N., Herrler T., Erichsen S., Schiergens T.S., Herrler G., Wu N.H., Nitsche A. (2020). SARS-CoV-2 Cell Entry Depends on ACE2 and TMPRSS2 and Is Blocked by a Clinically Proven Protease Inhibitor. Cell.

[10] Zhou P., Yang X.L., Wang X.G., Hu B., Zhang L., Zhang W., Si H.R., Zhu Y., Li B., Huang C.L. (2020). A pneumonia outbreak associated with a new coronavirus of probable bat origin. Nature.

[11] Shulla A., Heald-Sargent T., Subramanya G., Zhao J., Perlman S., Gallagher T. (2011). A Transmembrane Serine Protease Is Linked to the Severe Acute Respiratory Syndrome Coronavirus Receptor and Activates Virus Entry. J. Virol..

[12] Zipeto D., Palmeira J.D.F., Arganaraz G.A., Arganaraz E.R. (2020). ACE2/ADAM17/TMPRSS2 Interplay May Be the Main Risk Factor for COVID-19. Front. Immunol..

[13] Schreiber B., Patel A., Verma A. (2020). Shedding Light on COVID-19: ADAM17 the Missing Link. Am. J. Ther..

[14] Solerte S.B., Di Sabatino A., Galli M., Fiorina P. (2020). Dipeptidyl peptidase-4 (DPP4) inhibition in COVID-19. Acta Diabetol..

[15] Bassendine M.F., Bridge S.H., McCaughan G.W., Gorrell M.D. (2020). COVID-19 and comorbidities: A role for dipeptidyl peptidase 4 (DPP4) in disease severity. J. Diabetes.

[16] Cui C., Huang C., Zhou W., Ji X., Zhang F., Wang L., Zhou Y., Cui Q. (2020). AGTR2, one possible novel key gene for the entry of SARS-CoV-2 into human cells. IEEE/ACM Trans Comput. Biol. Bioinform..

[17] De Sousa A.K., Magalhaes D.A., Ferreira J.D.S., Barbosa A. (2020). SARS-CoV-2-mediated encephalitis: Role of AT2R receptors in the blood-brain barrier. Med. Hypothes..

[18] Qiao J., Li W., Bao J., Peng Q., Wen D., Wang J., Sun B. (2020). The expression of SARS-CoV-2 receptor ACE2 and CD147, and protease TMPRSS2 in human and mouse brain cells and mouse brain tissues. Biochem. Biophys. Res. Commun..

[19] Qi F., Qian S., Zhang S., Zhang Z. (2020). Single cell RNA sequencing of 13 human tissues identify cell types and receptors of human coronaviruses. Biochem. Biophys. Res. Commun..

[20] Padmanabhan P., Desikan R., Dixit N.M. (2020). Targeting TMPRSS2 and Cathepsin B/L together may be synergistic against SARS-CoV-2 infection. PloS Comput. Biol..

[21] IstIfl I.E., SihoGlu Tepe A., SarikUrkc U.C., Tepe B. (2020). Interaction of certain monoterpenoid hydrocarbons with the receptor binding domain of 2019 novel coronavirus (2019-nCoV), transmembrane serine protease 2 (TMPRSS2), cathepsin B, and cathepsin L (CatB/L) and their pharmacokinetic properties. Turk. J. Biol..

[22] Ellul M.A., Benjamin L., Singh B., Lant S., Michael B.D., Easton A., Kneen R., Defres S., Sejvar J., Solomon T. (2020). Neurological associations of COVID-19. Lancet Neurol..

[23] Mao L., Jin H., Wang M., Hu Y., Chen S., He Q., Chang J., Hong C., Zhou Y., Wang D. (2020). Neurologic Manifestations of Hospitalized Patients with Coronavirus Disease 2019 in Wuhan, China. JAMA Neurol..

[24] Solomon I.H., Normandin E., Bhattacharyya S., Mukerji S.S., Keller K., Ali A.S., Adams G., Hornick J.L., Padera R.F., Sabeti P. (2020). Neuropathological Features of COVID-19. N. Engl. J. Med..

[25] Virhammar J., Kumlien E., Fällmar D., Frithiof R., Jackmann S., Sköld M.K., Kadir M., Frick J., Lindeberg J., Olivero-Reinius H. (2020). Acute necrotizing encephalopathy with SARS-CoV-2 RNA confirmed in cerebrospinal fluid. Neurology.

[26] Baig A.M., Khaleeq A., Ali U., Syeda H. (2020). Evidence of the COVID-19 Virus Targeting the CNS: Tissue Distribution, Host–Virus Interaction, and Proposed Neurotropic Mechanisms. ACS Chem. Neurosci..

[27] Daneman R., Prat A. (2015). The blood-brain barrier. Cold Spring Harb. Perspect. Biol..

[28] Li Y., Li M., Wang M., Zhou Y., Chang J., Xian Y., Wang D., Mao L., Jin H., Hu B. (2020). Acute cerebrovascular disease following COVID-19: A single center, retrospective, observational study. Stroke Vasc. Neurol..

[29] Li Z., Liu T., Yang N., Han D., Mi X., Li Y., Liu K., Vuylsteke A., Xiang H., Guo X. (2020). Neurological manifestations of patients with COVID-19: Potential routes of SARS-CoV-2 neuroinvasion from the periphery to the brain. Front. Med..

[30] Barbosa L.C., Gonçalves T.L., de Araujo L.P., Rosario L.V.D.O., Ferrer V.P. (2021). Endothelial cells and SARS-CoV-2: An intimate relationship. Vasc. Pharmacol..

[31] Liu F., Han K., Blair R., Kenst K., Qin Z., Upcin B., Wörsdörfer P., Midkiff C.C., Mudd J., Belyaeva E. (2021). SARS-CoV-2 Infects Endothelial Cells In Vivo and In Vitro. Front. Cell. Infect. Microbiol..

[32] Huertas A., Montani D., Savale L., Pichon J., Tu L., Parent F., Guignabert C., Humbert M. (2020). Endothelial cell dysfunction: A major player in SARS-CoV-2 infection (COVID-19). Eur. Respir. J..

[33] Buzhdygan T.P., DeOre B.J., Baldwin-Leclair A., Bullock T.A., McGary H.M., Khan J.A., Razmpour R., Hale J.F., Galie P.A., Potula R. (2020). The SARS-CoV-2 spike protein alters barrier function in 2D static and 3D microfluidic in-vitro models of the human blood-brain barrier. Neurobiol. Dis..

[34] Stins M.F., Badgera J., Kim K.S. (2001). Bacterial invasion and transcytosis in transfected human brain microvascular endothelial cells. Microb. Pathog..

[35] WHO Laboratory Biosafety Guidance Related to Coronavirus Disease (COVID-19): Interim Guidance. https://www.who.int/publications/i/item/WHO-WPE-GIH-2021.1.

[36] Matos A.D.R., Caetano B.C., Filho J.L.D.A., Martins J.S.C.D.C., de Oliveira M.G.P., Sousa T.d.C., Horta M.A.P., Siqueira M.M., Fernandez J.H. (2022). Identification of Hypericin as a Candidate Repurposed Therapeutic Agent for COVID-19 and Its Potential Anti-SARS-CoV-2 Activity. Front. Microbiol..

[37] Barreto-Vieira D.F., da Silva M.A.N., Garcia C.C., Miranda M.D., Matos A.D.R., Caetano B.C., Resende P.C., Motta F.C., Siqueira M.M., Girard-Dias W. (2021). Morphology and morphogenesis of SARS-CoV-2 in Vero-E6 cells. Memórias Do Inst. Oswaldo Cruz.

[38] Kim D., Langmead B., Salzberg S.L. (2015). HISAT: A fast spliced aligner with low memory requirements. Nat. Methods.

[39] Liao Y., Smyth G.K., Shi W. (2014). feature Counts: An efficient general purpose program for assigning sequence reads to genomic features. Bioinformatics.

[40] Gu Z., Eils R., Schlesner M. (2016). Complex heatmaps reveal patterns and correlations in multidimensional genomic data. Bioinformatics.

[41] Robinson M.D., McCarthy D.J., Smyth G.K. (2010). EdgeR: A Bioconductor package for differential expression analysis of digital gene expression data. Bioinformatics.

[42] Wu T., Hu E., Xu S., Chen M., Guo P., Dai Z., Feng T., Zhou L., Tang W., Zhan L. (2021). Clusterprofiler 4.0: A universal enrichment tool for interpreting omics data. Innovation.

[43] Kolberg L., Raudvere U., Kuzmin I., Vilo J., Peterson H. (2020). gprofiler2—An R package for gene list functional enrichment analysis and namespace conversion toolset g:Profiler. F1000Research.

[44] Walter W., Sánchez-Cabo F., Ricote M. (2015). GOplot: An R package for visually combining expression data with functional analysis. Bioinformatics.

[45] Corman V.M., Landt O., Kaiser M., Molenkamp R., Meijer A., Chu D.K.W., Bleicker T., Brünink S., Schneider J., Schmidt M.L. (2020). Detection of 2019 novel coronavirus (2019-nCoV) by real-time RT-PCR. Eurosurveillance.

[46] Won J., Lee S., Park M., Kim T.Y., Park M.G., Choi B.Y., Kim D., Chang H., Kim V.N., Lee V.N.K.A.C.J. (2020). Development of a Laboratory-safe and Low-cost Detection Protocol for SARS-CoV-2 of the Coronavirus Disease 2019 (COVID-19). Exp. Neurobiol..

[47] Valente A.J., Maddalena L.A., Robb E.L., Moradi F., Stuart J.A. (2017). A simple ImageJ macro tool for analyzing mitochondrial network morphology in mammalian cell culture. Acta Histochem..

[48] Otsu N. (1979). A threshold selection method from gray-level histograms. IEEE Trans. Syst. Man Cybern..

[49] Arganda-Carreras I., Fernández-González R., Muñoz-Barrutia A., Ortiz-De-Solorzano C. (2010). 3D reconstruction of histological sections: Application to mammary gland tissue. Microsc. Res. Tech..

[50] Lee T., Kashyap R., Chu C. (1994). Building Skeleton Models via 3-D Medial Surface Axis Thinning Algorithms. CVGIP Graph. Model. Image Process..

[51] Terryn C., Sellami M., Fichel C., Diebold M.-D., Gangloff S., Le Naour R., Polette M., Zahm J.-M. (2012). Rapid method of quantification of tight-junction organization using image analysis. Cytom. Part A.

[52] Jiang M., Kolehmainen P., Kakkola L., Maljanen S., Melén K., Smura T., Julkunen I., Österlund P. (2021). SARS-CoV-2 Isolates Show Impaired Replication in Human Immune Cells but Differential Ability to Replicate and Induce Innate Immunity in Lung Epithelial Cells. Microbiol. Spectr..

[53] Takata F., Nakagawa S., Matsumoto J., Dohgu S. (2021). Blood-Brain Barrier Dysfunction Amplifies the Development of Neuroinflammation: Understanding of Cellular Events in Brain Microvascular Endothelial Cells for Prevention and Treatment of BBB Dysfunction. Front. Cell. Neurosci..

[54] Mockenhaupt K., Gonsiewski A., Kordula T. (2021). RelB and Neuroinflammation. Cells.

[55] Presta M., Foglio E., Schuind A.C., Ronca R. (2018). Long Pentraxin-3 Modulates the Angiogenic Activity of Fibroblast Growth Factor-2. Front. Immunol..

[56] Estato V., Stipursky J., Gomes F., Mergener T.C., Frazão-Teixeira E., Allodi S., Tibiriçá E., Barbosa H.S., Adesse D. (2018). The Neurotropic Parasite Toxoplasma gondii Induces Sustained Neuroinflammation with Microvascular Dysfunction in Infected Mice. Am. J. Pathol..

[57] Giacomello M., Pyakurel A., Glytsou C., Scorrano L. (2020). The cell biology of mitochondrial membrane dynamics. Nat. Rev. Mol. Cell Biol..

[58] Han H., Tan J., Wang R., Wan H., He Y., Yan X., Guo J., Gao Q., Li J., Shang S. (2020). PINK 1 phosphorylates Drp1 S616 to regulate mitophagy-independent mitochondrial dynamics. EMBO Rep..

[59] Sanchez C.V., Theel E., Binnicker M., Toledano M., McKeon A. (2021). Autoimmune Encephalitis After SARS-CoV-2 Infection: Case Frequency, Findings, and Outcomes. Neurology.

[60] Paterson R.W., Brown R.L., Benjamin L., Nortley R., Wiethoff S., Bharucha T., Jayaseelan D.L., Kumar G., Raftopoulos R.E., Zambreanu L. (2020). The emerging spectrum of COVID-19 neurology: Clinical, radiological and laboratory findings. Brain.

[61] Brann D.H., Tsukahara T., Weinreb C., Lipovsek M., Van Den Berge K., Gong B., Chance R., Macaulay I.C., Chou H.-J., Fletcher R.B. (2020). Non-neuronal expression of SARS-CoV-2 entry genes in the olfactory system suggests mechanisms underlying COVID-19-associated anosmia. Sci. Adv..

[62] McCracken I.R., Saginc G., He L., Huseynov A., Daniels A., Fletcher S., Peghaire C., Kalna V., Andaloussi-Mäe M., Muhl L. (2021). Lack of Evidence of Angiotensin-Converting Enzyme 2 Expression and Replicative Infection by SARS-CoV-2 in Human Endothelial Cells. Circulation.

[63] Conde J.N., Schutt W.R., Gorbunova E.E., Mackow E.R. (2020). Recombinant ACE2 Expression Is Required for SARS-CoV-2 To Infect Primary Human Endothelial Cells and Induce Inflammatory and Procoagulative Responses. Mbio.

[64] Krasemann S., Haferkamp U., Pfefferle S., Woo M.S., Heinrich F., Schweizer M., Appelt-Menzel A., Cubukova A., Barenberg J., Leu J. (2022). The blood-brain barrier is dysregulated in COVID-19 and serves as a CNS entry route for SARS-CoV-2. Stem Cell Rep..

[65] Heuberger J., Trimpert J., Vladimirova D., Goosmann C., Lin M., Schmuck R., Mollenkopf H., Brinkmann V., Tacke F., Osterrieder N. (2021). Epithelial response to IFN-γ promotes SARS-CoV-2 infection. EMBO Mol. Med..

[66] Meuren L.M., Prestes E.B., Papa M.P., de Carvalho L.R.P., Mustafá Y.M., da Costa L.S., Da Poian A.T., Bozza M.T., Arruda L.B. (2022). Infection of Endothelial Cells by Dengue Virus Induces ROS Production by Different Sources Affecting Virus Replication, Cellular Activation, Death and Vascular Permeability. Front. Immunol..

[67] Leda A., Bertrand L., Andras I.E., El-Hage N., Nair M., Toborek M. (2019). Selective Disruption of the Blood–Brain Barrier by Zika Virus. Front. Microbiol..

[68] Mladinich M.C., Conde J.N., Schutt W.R., Sohn S.-Y., Mackow E.R. (2021). Blockade of Autocrine CCL5 Responses Inhibits Zika Virus Persistence and Spread in Human Brain Microvascular Endothelial Cells. Mbio.

[69] DeOre B.J., Tran K.A., Andrews A.M., Ramirez S.H., Galie P.A. (2021). SARS-CoV-2 Spike Protein Disrupts Blood–Brain Barrier Integrity via RhoA Activation. J. Neuroimmune Pharmacol..

[70] Hao S., Ning K., Kuz C.A., Vorhies K., Yan Z., Qiu J. (2020). Long-Term Modeling of SARS-CoV-2 Infection of In Vitro Cultured Polarized Human Airway Epithelium. Mbio.

[71] Rauti R., Shahoha M., Leichtmann-Bardoogo Y., Nasser R., Paz E., Tamir R., Miller V., Babich T., Shaked K., Ehrlich A. (2021). Effect of SARS-CoV-2 proteins on vascular permeability. Elife.

[72] Saras J., Heldin C. (1996). PDZ domains bind carboxy-terminal sequences of target proteins. Trends Biochem. Sci..

[73] Giepmans B.N., Moolenaar W.H. (1998). The gap junction protein connexin43 interacts with the second PDZ domain of the zona occludens-1 protein. Curr. Biol..

[74] Dasgupta S., Bandyopadhyay M. (2021). Molecular docking of SARS-CoV-2 Spike epitope sequences identifies heterodimeric peptide-protein complex formation with human Zo-1, TLR8 and brain specific glial proteins. Med. Hypotheses.

[75] Caillet-Saguy C., Wolff N. (2021). PDZ-Containing Proteins Targeted by the ACE2 Receptor. Viruses.

[76] Feng S., Zou L., Wang H., He R., Liu K., Zhu H. (2018). RhoA/ROCK-2 Pathway Inhibition and Tight Junction Protein Upregulation by Catalpol Suppresses Lipopolysaccaride-Induced Disruption of Blood-Brain Barrier Permeability. Molecules.

[77] Mariano C., Palmela I., Pereira P., Fernandes A., Falcão A., Cardoso F.L., Vaz A.R., Campos A.R., Ferreira A.J.D.C.G., Kim K.S. (2012). Tricellulin expression in brain endothelial and neural cells. Cell Tissue Res..

[78] Constant O., Barthelemy J., Bolloré K., Tuaillon E., Gosselet F., Chable-Bessia C., Merida P., Muriaux D., Van de Perre P., Salinas S. (2021). SARS-CoV-2 Poorly Replicates in Cells of the Human Blood-Brain Barrier Without Associated Deleterious Effects. Front. Immunol..

[79] Zhang L., Zhou L., Bao L., Liu J., Zhu H., Lv Q., Liu R., Chen W., Tong W., Wei Q. (2021). SARS-CoV-2 crosses the blood–brain barrier accompanied with basement membrane disruption without tight junctions alteration. Signal Transduct. Target. Ther..

[80] Sun S.-C. (2012). The noncanonical NF-κB pathway. Immunol. Rev..

[81] Wenzel J., Lampe J., Müller-Fielitz H., Schuster R., Zille M., Müller K., Krohn M., Körbelin J., Zhang L., Özorhan (2021). The SARS-CoV-2 main protease Mpro causes microvascular brain pathology by cleaving NEMO in brain endothelial cells. Nat. Neurosci..

[82] Shih V.F.-S., Davis-Turak J., Macal M., Huang J.Q., Ponomarenko J., Kearns J.D., Yu T., Fagerlund R., Asagiri M., Zuniga I.E. (2012). Control of RelB during dendritic cell activation integrates canonical and noncanonical NF-κB pathways. Nat. Immunol..

[83] Chen X., El Gazzar M., Yoza B.K., McCall C.E. (2009). The NF-κB factor RelB and histone H3 lysine methyltransferase G9a directly interact to generate epigenetic silencing in endotoxin tolerance. J. Biol. Chem..

[84] Liu T.F., Yoza B.K., El Gazzar M., Vachharajani V., McCall C.E. (2011). NAD+-dependent SIRT1 Deacetylase Participates in Epigenetic Reprogramming during Endotoxin Tolerance. J. Biol. Chem..

[85] Coit P., De Lott L.B., Nan B., Elner V.M., Sawalha A.H. (2015). DNA methylation analysis of the temporal artery microenvironment in giant cell arteritis. Ann. Rheum. Dis..

[86] Li Y., Gorelik G., Strickland F.M., Richardson B.C. (2014). Oxidative Stress, T Cell DNA Methylation, and Lupus. Arthritis Rheumatol..

[87] Oaks Z., Perl A. (2013). Metabolic control of the epigenome in systemic Lupus erythematosus. Autoimmunity.

[88] Sawalha A.H., Zhao M., Coit P., Lu Q. (2020). Epigenetic dysregulation of ACE2 and interferon-regulated genes might suggest increased COVID-19 susceptibility and severity in lupus patients. Clin. Immunol..

[89] Corley M.J., Pang A.P., Dody K., Mudd P.A., Patterson B.K., Seethamraju H., Bram Y., Peluso M.J., Torres L., Iyer N.S. (2021). Genome-wide DNA methylation profiling of peripheral blood reveals an epigenetic signature associated with severe COVID-19. J. Leukoc. Biol..

[90] Mantovani A., Netea M.G. (2020). Trained Innate Immunity, Epigenetics, and COVID-19. N. Engl. J. Med..

[91] Ranjbar M., Rahimi A., Baghernejadan Z., Ghorbani A., Khorramdelazad H. (2022). Role of CCL2/CCR2 axis in the pathogenesis of COVID-19 and possible Treatments: All options on the Table. Int. Immunopharmacol..

[92] Schultheiß C., Willscher E., Paschold L., Gottschick C., Klee B., Bosurgi L., Dutzmann J., Sedding D., Frese T., Girndt M. (2022). Liquid biomarkers of macrophage dysregulation and circulating spike protein illustrate the biological heterogeneity in patients with post-acute sequelae of COVID-19. J. Med. Virol..

[93] Gedda M.R., Danaher P., Shao L., Ongkeko M., Chen L., Dinh A., Sall M.T., Reddy O.L., Bailey C., Wahba A. (2022). Longitudinal transcriptional analysis of peripheral blood leukocytes in COVID-19 convalescent donors. J. Transl. Med..

[94] Amer O.E., Sabico S., Sheshah E., Alotaibi N.H., Aldisi D.A., Enani M.A., Aljohani N.J., Alshingetti N., Alomar S.Y., Hussain S.D. (2022). Evaluation of 34 Cytokines and Vitamin D Status Reveal A Sexually-Dimorphic Active Immune Response to SARS-CoV-2. Healthcare.

[95] Alfadda A.A., Rafiullah M., Alkhowaiter M., Alotaibi N., Alzahrani M., Binkhamis K., Siddiqui K., Youssef A., Altalhi H., Almaghlouth I. (2022). Clinical and biochemical characteristics of people experiencing post-coronavirus disease 2019-related symptoms: A prospective follow-up investigation. Front. Med..

[96] Liu K.K., Dorovini-Zis K. (2012). Differential Regulation of CD4+ T Cell Adhesion to Cerebral Microvascular Endothelium by the β-Chemokines CCL2 and CCL3. Int. J. Mol. Sci..

[97] Eugenin E.A., Osiecki K., Lopez L., Goldstein H., Calderon T.M., Berman J.W. (2006). CCL2/Monocyte Chemoattractant Protein-1 Mediates Enhanced Transmigration of Human Immunodeficiency Virus (HIV)-Infected Leukocytes across the Blood–Brain Barrier: A Potential Mechanism of HIV–CNS Invasion and NeuroAIDS. J. Neurosci..

[98] Dhillon N.K., Williams R., Callen S., Zien C., Narayan O., Buch S. (2008). Roles of MCP-1 in development of HIV-dementia. Front. Biosci..

[99] Daigo K., Mantovani A., Bottazzi B. (2014). The yin-yang of long pentraxin PTX3 in inflammation and immunity. Immunol. Lett..

[100] Muffley L.A., Pan S.-C., Smith A.N., Ga M., Hocking A.M., Gibran N.S. (2012). Differentiation state determines neural effects on microvascular endothelial cells. Exp. Cell Res..

[101] Freese C., Hanada S., Fallier-Becker P., Kirkpatrick C.J., Unger R.E. (2017). Identification of neuronal and angiogenic growth factors in an in vitro blood-brain barrier model system: Relevance in barrier integrity and tight junction formation and complexity. Microvasc. Res..

[102] Shindo A., Maki T., Mandeville E.T., Liang A.C., Egawa N., Itoh K., Itoh N., Borlongan M., Holder J.C., Chuang T.T. (2016). Astrocyte-Derived Pentraxin 3 Supports Blood–Brain Barrier Integrity Under Acute Phase of Stroke. Stroke.

[103] Wesley U.V., Sutton I., Clark P.A., Cunningham K., Larrain C., Kuo J.S., Dempsey R.J. (2021). Enhanced expression of pentraxin-3 in glioblastoma cells correlates with increased invasion and IL8-VEGF signaling axis. Brain Res..

[104] Siqueira M., Francis D., Gisbert D., Gomes F.C.A., Stipursky J. (2017). Radial Glia Cells Control Angiogenesis in the Developing Cerebral Cortex Through TGF-β1 Signaling. Mol. Neurobiol..

[105] Breviario F., D’Aniello E.M., Golay J.T., Peri G., Bottazzi B., Bairoch A., Saccone S., Marzella R., Predazzi V., Rocchi M. (1992). Interleukin-1-inducible genes in endothelial cells. Cloning of a new gene related to C-reactive protein and serum amyloid P component. J. Biol. Chem..

[106] Fazzini F., Peri G., Doni A., Dell’Antonio G., Cin E.D., Bozzolo E., D’Auria F., Praderio L., Ciboddo G., Sabbadini M.G. (2001). PTX3 in small-vessel vasculitides: An independent indicator of disease activity produced at sites of inflammation. Arthritis Rheum..

[107] Rusnati M., Camozzi M., Moroni E., Bottazzi B., Peri G., Indraccolo S., Amadori A., Mantovani A., Presta M. (2004). Selective recognition of fibroblast growth factor-2 by the long pentraxin PTX3 inhibits angiogenesis. Blood.

[108] Meini S., Giani T., Tascini C. (2020). Intussusceptive angiogenesis in COVID-19: Hypothesis on the significance and focus on the possible role of FGF2. Mol. Biol. Rep..

[109] Ackermann M., Mentzer S.J., Kolb M., Jonigk D. (2020). Inflammation and intussusceptive angiogenesis in COVID-19: Everything in and out of flow. Eur. Respir. J..

[110] Mentzer S.J., Ackermann M., Jonigk D. (2022). Endothelialitis, Microischemia, and Intussusceptive Angiogenesis in COVID-19. Cold Spring Harb. Perspect. Med..

[111] Maldonado F., Morales D., Díaz-Papapietro C., Valdés C., Fernandez C., Valls N., Lazo M., Espinoza C., González R., Gutiérrez R. (2022). Relationship Between Endothelial and Angiogenesis Biomarkers Envisage Mortality in a Prospective Cohort of COVID-19 Patients Requiring Respiratory Support. Front. Med..

[112] Gopinathan G., Milagre C., Pearce O.M., Reynolds L.E., Hodivala-Dilke K., Leinster D.A., Zhong H., Hollingsworth R.E., Thompson R., Whiteford J.R. (2015). Interleukin-6 Stimulates Defective Angiogenesis. Cancer Res..

[113] Reyes A., Corrales N., Gálvez N.M.S., Bueno S.M., Kalergis A.M., González P.A. (2020). Contribution of hypoxia inducible factor-1 during viral infections. Virulence.

[114] Ke Q., Costa M. (2006). Hypoxia-inducible factor-1 (HIF-1). Mol. Pharmacol..

[115] Afsar B., Kanbay M., Afsar R.E. (2020). Hypoxia inducible factor-1 protects against COVID-19: A hypothesis. Med. Hypotheses.

[116] Zhang R., Wu Y., Zhao M., Liu C., Zhou L., Shen S., Liao S., Yang K., Li Q., Wan H. (2009). Role of HIF-1α in the regulation ACE and ACE2 expression in hypoxic human pulmonary artery smooth muscle cells. Am. J. Physiol. Cell. Mol. Physiol..

[117] Imperio G.E., Lye P., Mughis H., Hamada H., Bloise E., Lye S.J., Matthews S.G. (2021). Hypoxia alters the expression of ACE2 and TMPRSS2 SARS-CoV-2 cell entry mediators in hCMEC/D3 brain endothelial cells. Microvasc. Res..

[118] Walmsley S.R., Print C., Farahi N., Peyssonnaux C., Johnson R.S., Cramer T., Sobolewski A., Condliffe A.M., Cowburn A.S., Johnson N. (2005). Hypoxiainduced neutrophil survival is mediated by HIF-1αdependent NF-κB activity. J. Exp. Med..

[119] Oldendorf W.H., Cornford M.E., Brown W.J. (1977). The large apparent work capability of the blood-brain barrier: A study of the mitochondrial content of capillary endothelial cells in brain and other tissues of the rat. Ann. Neurol..

[120] Parodi-Rullán R.M., Javadov S., Fossati S. (2021). Dissecting the Crosstalk between Endothelial Mitochondrial Damage, Vascular Inflammation, and Neurodegeneration in Cerebral Amyloid Angiopathy and Alzheimer’s Disease. Cells.

[121] Doll D.N., Hu H., Sun J., Lewis S.E., Simpkins J.W., Ren X. (2015). Mitochondrial Crisis in Cerebrovascular Endothelial Cells Opens the Blood–Brain Barrier. Stroke.

[122] Shi C.-S., Qi H.-Y., Boularan C., Huang N.-N., Abu-Asab M., Shelhamer J.H., Kehrl J.H. (2014). SARS-Coronavirus Open Reading Frame-9b Suppresses Innate Immunity by Targeting Mitochondria and the MAVS/TRAF3/TRAF6 Signalosome. J. Immunol..

[123] Romão P.R.T., Teixeira P.C., Schipper L., da Silva I., Filho P.S., Júnior L.C.R., Peres A., da Fonseca S.G., Monteiro M.C., Lira F.S. (2022). Viral load is associated with mitochondrial dysfunction and altered monocyte phenotype in acute severe SARS-CoV-2 infection. Int. Immunopharmacol..

[124] Lei Y., Zhang J., Schiavon C.R., He M., Chen L., Shen H., Zhang Y., Yin Q., Cho Y., Andrade L. (2021). SARS-CoV-2 Spike Protein Impairs Endothelial Function via Downregulation of ACE 2. Circ. Res..

[125] Di Domizio J., Gulen M.F., Saidoune F., Thacker V.V., Yatim A., Sharma K., Nass T., Guenova E., Schaller M., Conrad C. (2022). The cGAS–STING pathway drives type I IFN immunopathology in COVID-19. Nature.

[126] Forrester S.J., Preston K.J., Cooper H.A., Boyer M.J., Escoto K.M., Poltronetti A.J., Elliott K.J., Kuroda R., Miyao M., Sesaki H. (2020). Mitochondrial Fission Mediates Endothelial Inflammation. Hypertension.

[127] Jendrach M., Pohl S., Vöth M., Kowald A., Hammerstein P., Bereiter-Hahn J. (2005). Morpho-dynamic changes of mitochondria during ageing of human endothelial cells. Mech. Ageing Dev..

[128] Burns E.M., Kruckeberg T.W., Comerford L.E., Buschmann M.T. (1979). Thinning of Capillary Walls and Declining Numbers of Endothelial Mitochondria in the Cerebral Cortex of the Aging Primate, Macaca Nemestrina. J. Gerontol..

[129] Shenouda S.M., Widlansky M.E., Chen K., Xu G., Holbrook M., Tabit C.E., Hamburg N.M., Frame A.A., Caiano T.L., Kluge M.A. (2011). Altered Mitochondrial Dynamics Contributes to Endothelial Dysfunction in Diabetes Mellitus. Circulation.

[130] Wang Z., Liu Y., Liu J., Liu K., Wen J., Wen S., Wu Z. (2011). HSG/Mfn2 Gene Polymorphism and Essential Hypertension: A Case-Control Association Study in Chinese. J. Atheroscler. Thromb..

[131] Twig G., Hyde B., Shirihai O.S. (2008). Mitochondrial fusion, fission and autophagy as a quality control axis: The bioenergetic view. Biochim. Biophys. Acta BBA Bioenerg..

[132] Sugiura A., McLelland G.-L., Fon E.A., McBride H.M. (2014). A new pathway for mitochondrial quality control: Mitochondrial-derived vesicles. EMBO J..

[133] Picca A., Guerra F., Calvani R., Coelho-Junior H.J., Bossola M., Landi F., Bernabei R., Bucci C., Marzetti E. (2020). Generation and Release of Mitochondrial-Derived Vesicles in Health, Aging and Disease. J. Clin. Med..

[134] Zhong Z., Umemura A., Sanchez-Lopez E., Liang S., Shalapour S., Wong J., He F., Boassa D., Perkins G., Ali S.R. (2016). NF-κB Restricts Inflammasome Activation via Elimination of Damaged Mitochondria. Cell.

[135] Nakahira K., Haspel J.A., Rathinam V.A.K., Lee S.-J., Dolinay T., Lam H.C., Englert J.A., Rabinovitch M., Cernadas M., Kim H.P. (2011). Autophagy proteins regulate innate immune responses by inhibiting the release of mitochondrial DNA mediated by the NALP3 inflammasome. Nat. Immunol..

[136] Zhou R., Yazdi A.S., Menu P., Tschopp J. (2011). A role for mitochondria in NLRP3 inflammasome activation. Nature.

[137] Liu Q., Zhang D., Hu D., Zhou X., Zhou Y. (2018). The role of mitochondria in NLRP3 inflammasome activation. Mol. Immunol..

