# Supplementary material for: Human Brain Microvascular Endothelial Cells Exposure to SARS-CoV-2 Leads to Inflammatory Activation through NF-κB Non-Canonical Pathway and Mitochondrial Remodeling
Source: Viruses. 2023 Mar 14;15(3):745. doi: 10.3390/v15030745 (PMC10056985; doi:10.3390/v15030745)
Supplement: Supplementary file 1 [file viruses-15-00745-s001.zip › viruses-2176478-supplementary.pdf]

Table S1. RT-qPCR primer sequences used in this work.

Gene name	Forward primer (5′-3′)	Reverse Primer (5′-3′)
ACE2	TCCATTGGTCTTCTGTCACCCG	AGACCATCCACCTCCACTTCTC
Claudin-5	GTGTCGCAGCAGAAGTACGAG	ACGTGTCCTCCTTGTTTAAG
Drp1	GATGCCATAGTTGAAGTGGTGAC	CCACAAGCATCAGCAAAGTCTGG
Fis1	CAAGGAACTGGAGCGGCTCATT	GGACACAGCAAGTCCGATGAGT
IF-1α	TATGAGCCAGAAGAACTTTTAGGC	CACCTCTTTTGGCAAGCATCCTG
MFF	CAA GGT TCC AGG CAC CGA TTT C	GCG ACÇA AAA TGC CAC GAG CAG A
Mfn2	ATT GCA GAG GCG GTT CGA CTC A	TTC AGT CGG TCT TGC CGC TCT T
SARS-CoV-2 Spike1	CTACATGCACCAGCAACTGT	CACCTGTGCCTGTTAAACCA
TOMM20	CGA CCG CAA AAG ACG AAG TGA C	GCT TCA GCA TCT TTA AGG TCA GG
ZO- 1	ACCAGTAAGTCGTCCTGATCC	TCGGCCAAATCTTCTCACTCC

Table S2. Antibodies used in this work.

Target name	Company name	Reference	Host	Dilution
TOMM20 (immunofluorescence)	Thermo Fisher Scientific	MA5 24859	Rabbit	1:200
TOMM20 (western blotting)	Cell Signaling Technologies	PA5-52843	Rabbit	1:1,000
ZO-1	Thermo Fisher Scientific	33-9100	Mouse	1:200
Claudin-5	Thermo Fisher Scientific	35-2500	Mouse	1:1,000
Cleaved caspase3	Cell Signaling Technologies	9661S	Rabbit	1:200
Spike1	Thermo Fisher Scientific	703971	Rabbit	1 :100
TMPRSS2	Thermo Fisher Scientific	PA5-14264	Rabbit	1:1,000
ACE2	ABCAM	ab15348	Rabbit	1:1,000
MFF	Cell Signaling Technologies	86668s	Rabbit	1:1,000
Fis1	ABCAM	ab156865	Rabbit	1:1,000
Drp1	Cell Signaling Technologies	14647s	Mouse	1:1,000
phosphoDrp1 (S616)	Cell Signaling Technologies	#3455	Rabbit	1:1,000
Mitofusin2 (Mfn2)	Cell Signaling Technologies	11925T	Rabbit	1:1,000
AlexaFluor 546 anti-mouse IgG (H+L)	Thermo Fisher Scientific	A11003	Goat	1:1,000
AlexaFluor 488 – anti-rabbit IgG (H+L)	Thermo Fisher Scientific	A32790	Donkey	1:1,000
